# Multi-state models of transitions in depression and anxiety symptom severity and cardiovascular events in patients with coronary heart disease

**DOI:** 10.1371/journal.pone.0213334

**Published:** 2019-03-07

**Authors:** Michelle L. Meyer, Feng-Chang Lin, Andrea Jaensch, Ute Mons, Harry Hahmann, Wolfgang Koenig, Hermann Brenner, Dietrich Rothenbacher

**Affiliations:** 1 Department of Emergency Medicine, University of North Carolina at Chapel Hill, Chapel Hill, North Carolina, United States of America; 2 Department of Biostatistics, University of North Carolina at Chapel Hill, Chapel Hill, North Carolina, United States of America; 3 Institute of Epidemiology and Medical Biometry, Ulm University, Ulm, Germany; 4 Division of Clinical Epidemiology and Aging Research, German Cancer Research Center (DKFZ), Heidelberg, Germany; 5 Klinik Schwabenland, Isny-Neutrauchburg, Germany; 6 Department of Internal Medicine II-Cardiology, University of Ulm Medical Center, Ulm, Germany; 7 Deutsches Herzzentrum München, Technische Universität München, Munich, Germany; 8 German Centre for Cardiovascular Research (DZHK), partner site Munich Heart Alliance, Munich, Germany; 9 Division of Preventive Oncology, German Cancer Research Center (DKFZ) and National Center for Tumor Diseases (NCT), Heidelberg, Germany; Nathan S Kline Institute, UNITED STATES

## Abstract

**Objective:**

Patients with coronary heart disease (CHD) commonly suffer from depression and anxiety, yet transitions of symptom severity and cardiovascular events (CVE) over time are not well characterized.

**Methods:**

We included 997 patients with stable CHD from a prospective cohort study. We estimated 5- and 10-year transition probabilities of depression and anxiety symptom severity levels and fatal- and non-fatal adverse CVE. Depression and anxiety symptoms were measured with the Hospital Anxiety and Depression Scale 5 times over 13 years and categorized as no, mild, or moderate/severe symptoms. Using multi-state modeling, we calculated 5- and 10-year transition probabilities for depression and anxiety symptom severity and CVE and calculated transition intensity ratios for factors associated with symptom severity progression and regression.

**Results:**

At 5 years, only approximately half of participants with moderate or severe symptom severity at baseline transitioned to no symptom severity. Patients with low physical activity (<1x/week or never) had a higher probability of worse symptom severity after 5 and 10 years and a higher probability of a CVE after 5 and 10 years regardless of their depression status at baseline compared to higher physical activity groups. Higher body mass index, <10 years of education, and lower physical activity were associated with depression symptom progression; female and lower physical activity were associated with anxiety symptom progression.

**Conclusions:**

Patients with CHD had a consistent burden of depression and anxiety symptoms. Secondary prevention strategies should target depression and anxiety and include a physical activity component.

## Introduction

Coronary heart disease (CHD) is a leading cause of morbidity and mortality worldwide [1]. Individuals with known CHD commonly report symptoms of depression [2, 3] and anxiety [4], which are associated with a worse prognosis. Although screening for mood disorders is recommended for secondary prevention of adverse cardiovascular events among those with CHD [5–9], it is not commonly performed in clinical routine.

Meta-analyses of studies among individuals with CHD show that symptoms of depression [2, 3] and anxiety [4] are associated with the risk of fatal- and non-fatal adverse cardiovascular events (CVE) and all-cause mortality. There is increasing evidence that health behavior, particularly physical activity, reduces depression and anxiety as well as mortality after a cardiovascular event [10–13]. Most studies evaluated baseline or short-term changes in symptoms of depression and anxiety with prognosis in individuals with CHD and did not consider the role of physical activity.

Transitions of depression and anxiety symptom severity over a course of many years have not been well characterized in individuals with CHD and their association with long-term prognostic outcomes has not been sufficiently examined. Understanding transitions in depression and anxiety symptom severity and factors associated with these transitions are key to develop interventions to improve long-term prognosis in individuals with CHD. We therefore characterized 5- and 10-year transitions of depression and anxiety symptom severity and CVE among participants with CHD in a prospective cohort study. Additionally, we evaluated factors associated with progression and regression of the severity of depression and anxiety symptoms. We hypothesized that depression and anxiety symptoms change over 10 years in individuals with CHD. Additionally, the burden of depression and anxiety symptoms will be higher among older individuals and those with lower physical activity levels, which would be associated with symptom progression.

## Materials and methods

### Study population and study design

The prospective KAROLA cohort study included participants with CHD (International Classification of Diseases (ICD), 9^th^ Rev. pos. 410–414) aged 30–70 years participating in an in-patient rehabilitation program between January 1999 and May 2000 in one of two rehabilitation clinics in Germany (Schwabenland-Klinik, Isny and Klinik am Südpark, Bad Nauheim), as previously described [14]. In Germany, all patients discharged from an acute care hospital after an acute coronary syndromes or coronary artery bypass grafting are offered a comprehensive in-hospital rehabilitation program.

KAROLA only included participants who were admitted within three months after their first acute event or coronary artery bypass grafting. Of all eligible participants admitted to the in-patient rehabilitation clinic during the recruitment period, 58% agreed to participate (n = 1206). In total, 997 participants with information on the Hospital Anxiety and Depression Scale (HADS) from end of in-patient rehabilitation and during follow-up were included in the final analysis. All subjects gave written informed consent. The study was approved by the Ethics Boards of the Universities of Ulm (No.186/98) and Heidelberg and the Physicians’ chambers of the States of Baden-Württemberg and Hessen.

### Data collection

Baseline characteristics were determined from an interview-based questionnaire within the in-patient rehabilitation clinic and taken from medical records at the beginning of the in-patient rehabilitation program. Information was also taken from the participants’ hospital charts. Leisure time physical activity was assessed during one year follow-up with the question “On average, how often have you engaged in physically strenuous and sweat-inducing activity in your leisure time in the past 12 months (i.e. cycling, speedy hiking, gardening, sport)?” and answers were categorized into “daily”, “5 to 6 times per week”, “2 to 4 times per week”, “1 to 4 times per month”, “rarely or never”.

### Assessment of anxiety and depressive symptoms

Anxiety and depressive symptoms were evaluated at baseline (end of in-patient rehabilitation), and at one, three, six, and eight year follow-up by the HADS in German [15]. HADS is a standardized, self-administered questionnaire containing 14 questions to quantify generalized anxiety and depression (7 items each) in medical patients [16] and performs well in patients with cardiac diseases. All items are scored on a four point Likert scale (0–3 points). A summary score is calculated for both the anxiety subscale and depression subscale, each ranging from 0 to 21. For both HADS subscales, a ≥8 cut-point is suggested as optimal to detect symptoms for anxiety disorders and depression [16]. For this analysis, we categorized both subscales as <8 “normal”, 8–10 “moderate”, and ≥ 11 “severe.”

### Follow-up and evaluation of cardiovascular disease events

For all participants, active follow-up was conducted one, three, four and a half, six, eight, ten, and thirteen years after discharge from the rehabilitation clinic. Information was obtained from the patient via a mailed standardized questionnaire. Up to December 24, 2013, primary care physicians completed standardized questionnaires regarding non-fatal adverse CVE and treatment since discharge from the in-patient rehabilitation clinic, including use of anti-depressant medication and psycholeptics (Anatomical Therapeutic Chemical Code N06A and N05, respectively). If a participant died during follow-up, the death certificate was obtained from local Public Health departments and the main cause of death was coded according to the International Classification of Diseases (ICD-9 pos. 390–459; ICD-10 pos. I0-I99 and R57.0). An adverse CVE was defined as cardiovascular disease as the main cause of death and primary care physician reported diagnosis of non-fatal myocardial infarction or stroke.

### Statistical analysis

**Fig 1:**
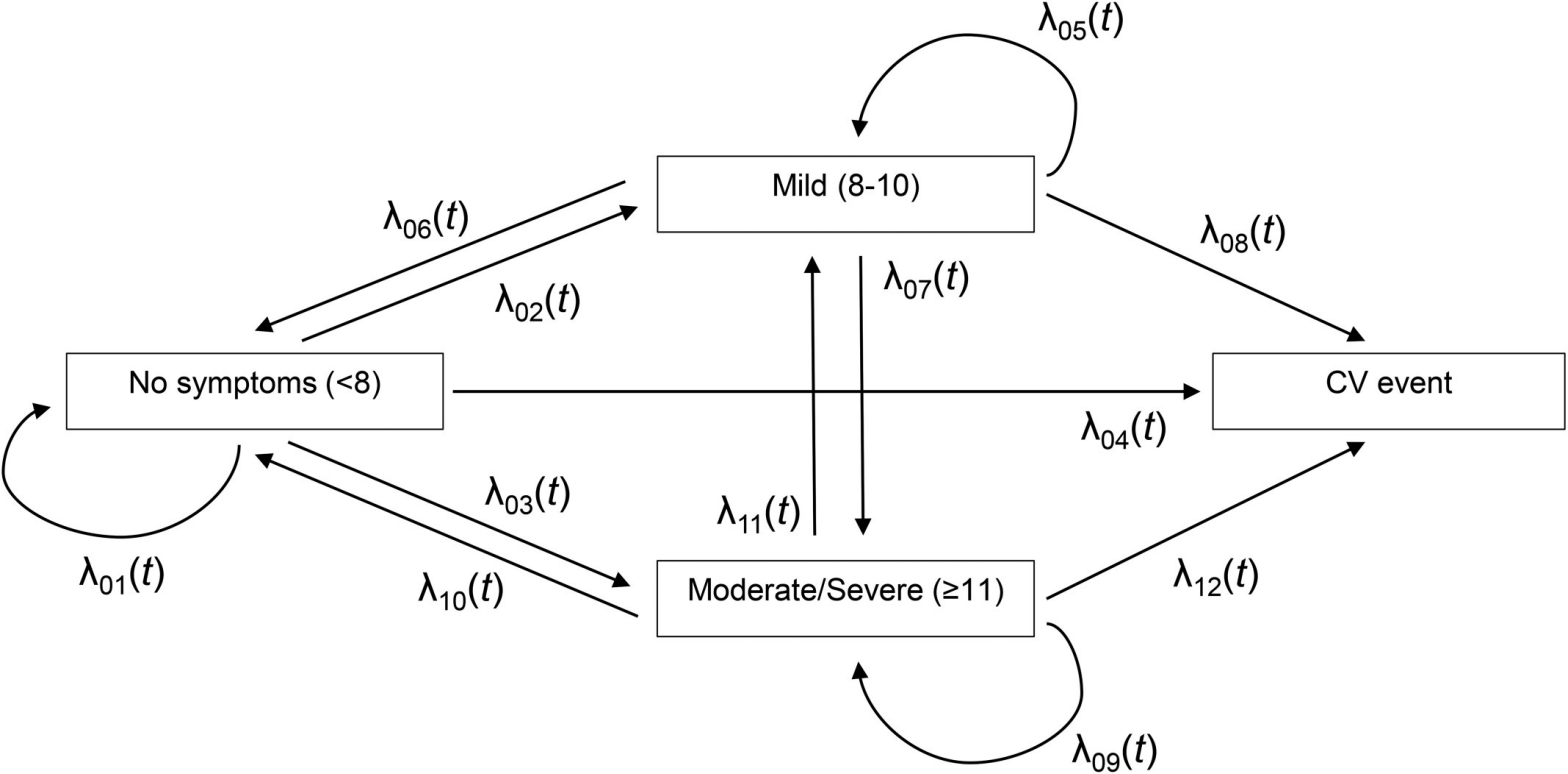
Multistate model of transitions in depression or anxiety symptom severity and a cardiovascular event (CVE).

Participant characteristics were described as means and standard deviations and frequencies and percentages, where appropriate. We used continuous-time multi-state models [17] to estimate 12 transition intensities, *λ_ij_*(*t*), between states of depression and anxiety symptom severity of 1) no symptoms, 2) mild, and 3) moderate/severe, as well as a subsequent CVE (Fig 1). For example, λ_02_(*5*) represents the instantaneous transition intensity or hazard rate of a transition from state no symptoms to state mild symptoms at 5 years. This multi-state model is an ‘illness-death model’ whereby symptom severity are transient states, allowing future transitions between each other whereas CVE is an absorbing state. The models were assumed to follow 1) Cox proportional intensity models with heterogeneous baseline intensity functions (i.e., each transition has its own baseline hazard), and 2) Markov process in which the future state depends on the history only through the present state (not on the preceding sequence of events).

Regression coefficients were estimated via a stratified partial likelihood without specifying any of the baseline functions. Transition probability predictions for severity of symptoms and CVE at 5 and 10 years from different ages and physical activity levels were estimated by a continuous product integral of the transition intensities [17] derived from the estimated regression coefficients and baseline intensity functions with adjustment for established cardiovascular risk factors from the literature such as sex, body mass index (BMI), smoking, education, diabetes, myocardial infarction, physical activity, and anti-depression/anxiety medications; transition probabilities are shown at the reference level. We conducted the same analysis for anxiety symptoms. To assess the dependence of the transition probabilities on age and physical activity, we stratified the analysis by age (<55 yrs., 55–64 yrs., and >64 yrs.) and physical activity (PA) level (PA level 1: daily or 5-6x/week, PA level 2: 2-4x/week, PA level 3: 1x/week or 1-3x/month, and PA level 4: seldom or never).

To show the effects of explanatory variables on depression or anxiety symptom progression, regression, and CVE, we calculated transitions separately for each covariate and used a likelihood ratio test to evaluate whether effects were the same or different within explanatory variables, e.g., age from level 1 (<55 yr) to 2 (55–64 yr) to 3 (>64 yr) as an ordinal scale for each transition. This results in transition-specific estimated regression coefficients, or a ratio of transition intensities (transition intensity ratios (TIR), and their 95% confidence intervals 95% CI). TIR is the ratio of transition intensities between the exposed group to the unexposed group and can be interpreted as relative risk (RR). TIRs were adjusted for age (<55 yrs., 55–64 yrs., and >64 yrs.), female sex, BMI (<25 kg/m^2^ normal, 25–29.9 kg/m^2^ overweight, and ≥30 kg/m^2^ obese), current/former smoker (vs. never), education <10 years (vs. education 10+), history of diabetes (yes vs. no), history of myocardial infarction (yes vs. no), lower physical activity at year one (PA level 1: daily or 5-6x/week, PA level 2: 2-4x/week, PA level 3: 1x/week or 1-3x/month, and PA level 4: seldom or never), and anti-depression/anxiety medication use. Categories of age, BMI, and physical activity were treated as ordinal variables where the TIR estimates are for every one increase in the category of the variable (compared to the lowest (reference) category). Analyses were conducted using SAS (version 9.4, SAS Institute, Inc., Cary, NC) and mstate package in R (R version 3.3.3).

## Results

The average age of the participants was 58.9 years and ranged from 30 to 70 years (Table 1). About 15% of the participants were women. The average BMI was 26.9 (range 18.1 to 44.2) kg/m^2^, over half of the participants had a history of a myocardial infarction, and 16.2% reported type 2 diabetes. The use of anti-depression or anti-anxiety medications was low at 2%. At baseline, 11.4% had mild and 5.6% had moderate/severe depression symptoms, and 15.7% had mild and 7.5% had moderate/severe anxiety symptoms.

**Table 1:** Demographic and clinical characteristics in patients with coronary heart disease (n = 997).

Characteristics at baseline	Mean (SD) or N (%)
Age, years, mean (SD)	58.9 (8.0)
Female, n (%)	150 (15.1)
Body mass index, Kg/m^2^, mean (SD)	26.9 (3.2)
School education <10 years	597 (59.9)
Married, n (%)	842 (84.5)
Former/current smoker, n (%)	667 (66.9)
Physical activity at year one, n (%)^a^	
PA level 1, daily or 5-6x/week	310 (31.2)
PA level 2, 2-4x/week	417 (42.0)
PA level 3, 1x/week or 1-3x/month	180 (18.1)
PA level 4, seldom or never	87 (8.8)
History of myocardial infarction, n (%)	570 (57.2)
History of type 2 diabetes, n (%)	161 (16.2)
Clinical score (angiographic evaluation)^b^	
0 or 1 vessel disease	259 (27.2)
2 vessel disease	269 (28.3)
3 vessel disease	423 (44.5)
Medication use, n (%)^c^	
Anti-depression	20 (2.0)
Anti-anxiety	18 (1.8)
Beta‐blocker	873 (87.7)
ACE‐inhibitor	519 (52.2)
Lipid-lowering	761 (76.5)
HADS-depression score, n (%)	
<8, no symptoms	827 (82.9)
8‐10, mild symptoms	114 (11.4)
≥11, moderate/severe symptoms	56 (5.6)
HADS-anxiety score, n (%)	
<8, no symptoms	765 (76.7)
8‐10, mild symptoms	157 (15.7)
≥11, moderate/severe symptoms	75 (7.5)

SD, standard deviation; PA, physical activity; HADS, Hospital Anxiety and Depression Scale.

^a^3 missing

^b^46 missing

^c^2 missing.

### Transitions in depression and anxiety symptom severity by age groups

**Fig 2:**
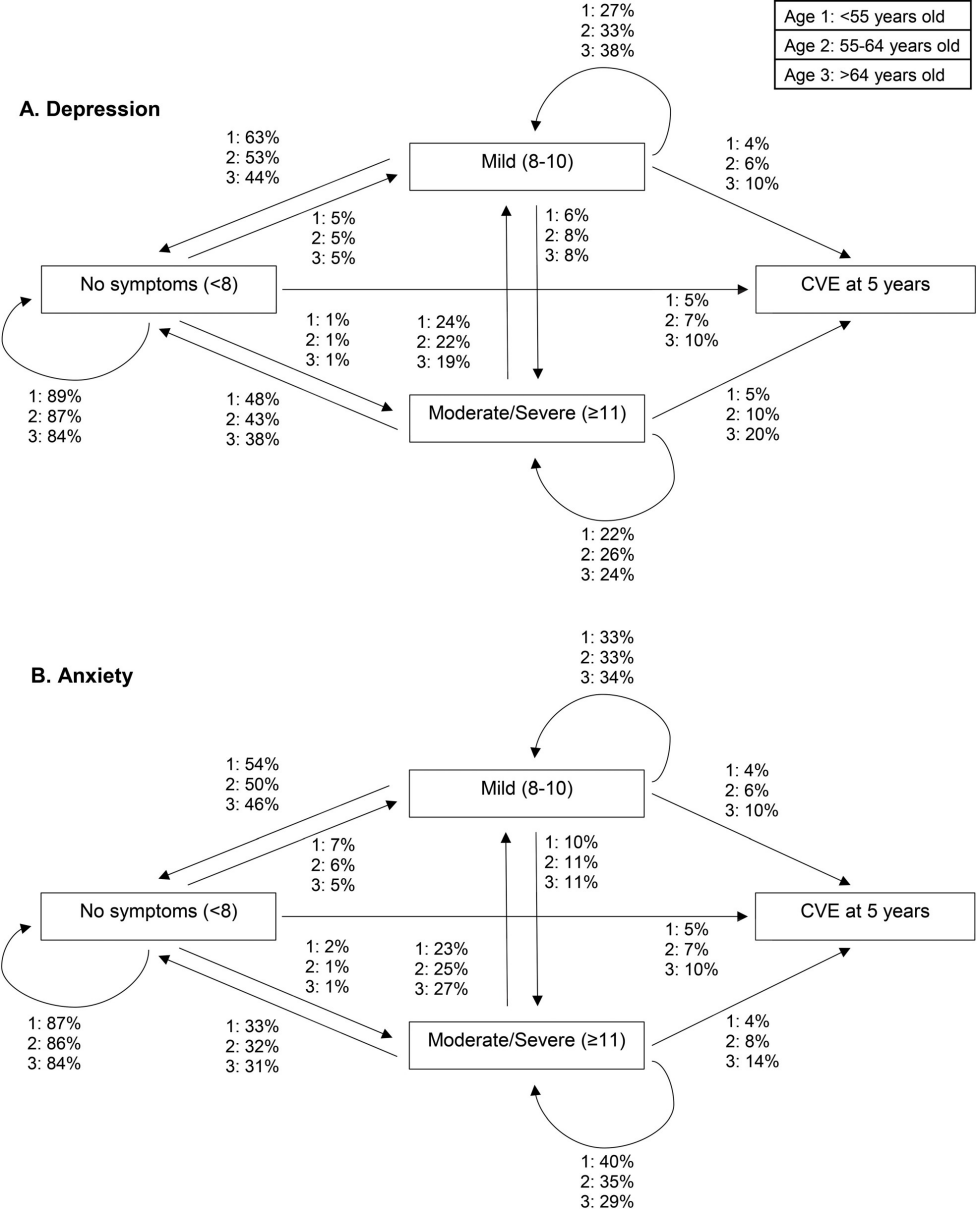
Transitions in (A) depression and (B) anxiety symptom severity and a cardiovascular event (CVE) at 5 years by age group with adjustments for sex, body mass index, smoking, education, diabetes, myocardial infarction, physical activity and anti-depression/anxiety medication; transition probabilites are shown at the reference level, N = 997.

Participants with no depression symptoms at baseline were more likely to stay at the same level at 5 years (range 84–89% in the three different age categories; Fig 2A; Table A in S2 File). In addition, at 5 years, 44–63% of participants with mild and 38–48% of participants with moderate/severe depression symptoms at baseline transitioned to no symptoms, yet 27–38% of participants with mild and 22–26% of participants with moderate/severe depression symptoms stayed at the same level in the respective age categories. In general, older participants were less likely than younger participants to transition to no depression symptoms. As displayed in Fig A in S1 File and Table A in S2 File, at 10 years, participants >64 years old were less likely to transition to no symptoms and were more likely to transition to a CVE compared to those <55 and 55–64 years old.

Participants with no anxiety symptoms at baseline were more likely to stay at the same level at 5 years (range 84–87%; Fig 2B; Table B in S2 File). Furthermore, at 5 years, 46–54% of participants with mild and 31–33% of participants with moderate/severe anxiety symptoms transition to no symptoms. Still, at 5 years, 33–34% of participants with mild and 29–40% of moderate/severe symptoms of anxiety stayed at the same severity level. As displayed in Fig A in S1 File and Table B in S2 File, at 10 years, 14% of participants with mild and 5–13% of participants with moderate/severe anxiety symptoms at baseline remained at the same symptom severity level.

For both depression and anxiety symptoms (Fig 2, Fig A in S1 File, and Tables A and B in S2 File), the probability of a CVE at 5 and 10 years increased with age. Notably, those in the age category >64 and with moderate/severe depression symptoms had much higher transition probabilities to CVE than the same age group without symptoms at baseline (19.8% vs. 9.8% at 5 years; 40.3% vs. 23.7% at 10 years, respectively).

### Transitions in depression and anxiety symptom severity by physical activity level

**Fig 3:**
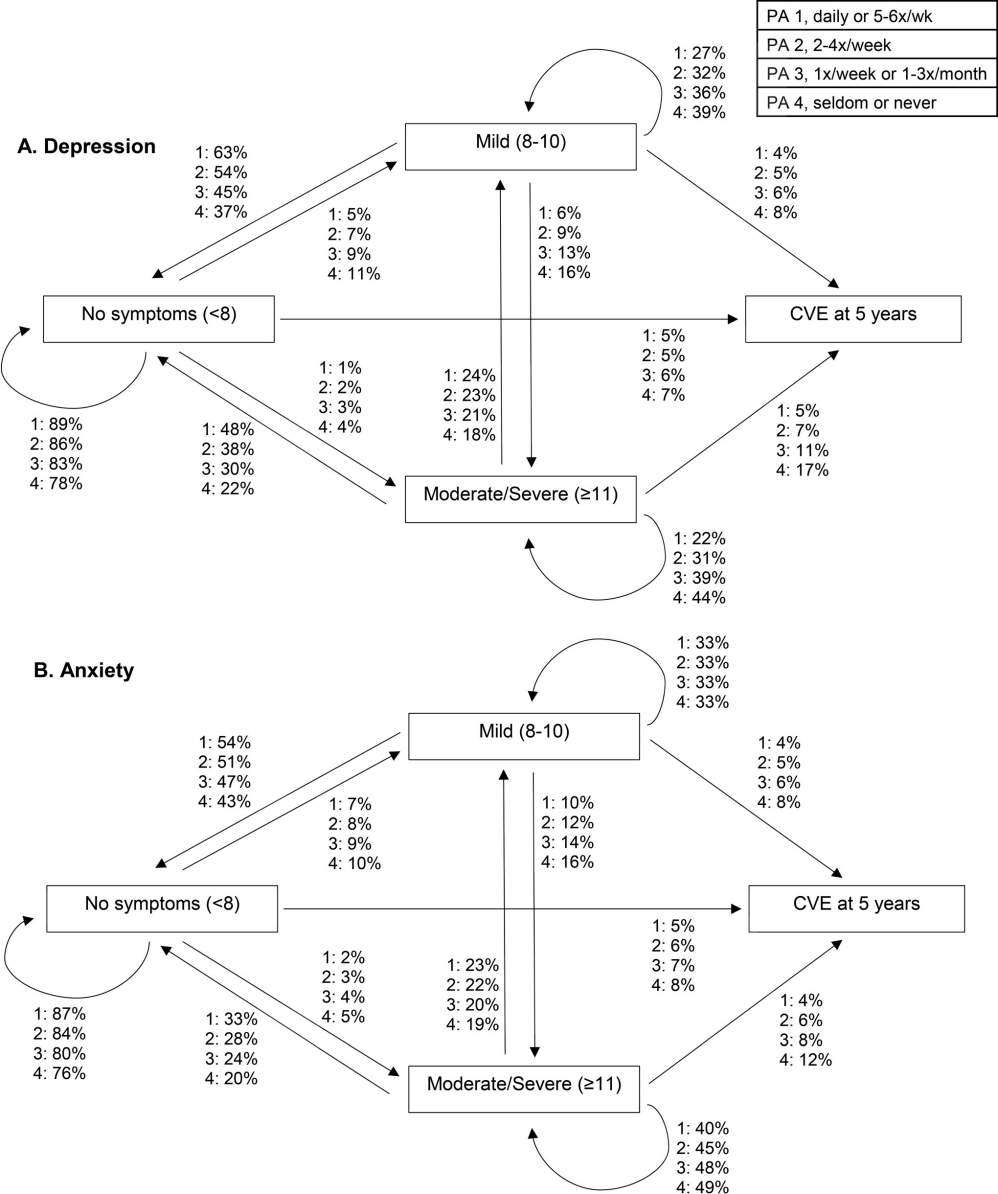
Transitions in (A) depression and (B) anxiety symptom severity and a cardiovascular event (CVE) at 5 years by physical activity (PA) group with adjustments for sex, body mass index, smoking, education, diabetes, myocardial infarction, physical activity and anti-depression/anxiety medication; transition probabilites are shown at the reference level, N = 997.

At 5 years, 37–63% of participants with mild and 22–48% of participants with moderate/severe depression symptoms transitioned to no symptoms, with lower physical activity groups having a lower transition to no symptoms (Fig 3A; Table C in S2 File). Among those with moderate/severe depression symptoms at baseline, 44% of those in the lowest physical activity group remained at moderate/severe symptoms compared to 22% in the highest physical activity group at 5 years. A similar pattern was observed at 10 years (Fig B in S1 File and Table C in S2 File).

The results for anxiety were similar to those for depression, but the association with physical activity levels was weaker as the transition probabilities were similar across physical activity levels (Fig 3B; Table D in S2 File). Among participants with moderate/severe anxiety symptoms in the lowest two physical activity levels, 48 and 49% were more likely to stay at the same level at 5 years, respectively, and only 20 and 24% transitioned to no symptoms at 5 years. At 10 years, participants in the lowest two physical activity groups were more likely to transition to a CVE compared to higher physical activity groups (Fig B in S1 File and Table D in S2 File).

### Associations of explanatory variables on depression and anxiety progression, regression, and CVE

As shown in Table 2, factors associated with the progression of depression symptoms were higher BMI categories (TIR_1→ 2_: 1.3), <10 years of education (TIR_1→ 2_: 1.4; TIR_1→ 3_: 1.9), and lower physical activity groups (TIR_1→ 2_: 1.3; TIR_1→ 3_:1.6). Factors associated with a lower risk of regression in depressive symptoms included older age groups (TIR_2→1_: 0.8), higher BMI categories (TIR_2→1_: 0.7), history of a myocardial infarction (TIR_3→2_: 0.6), and lower physical activity groups (TIR_2→1_: 0.8; TIR_3→2_: 0.7). Older age groups were associated with a higher risk of a CVE and the association was stronger with increasing depression levels. Other factors associated with a CVE by depression levels included the following: female sex (TIR_1→CVE_: 0.5), <10 years of education (TIR_3→CVE_: 0.4), and history of diabetes (TIR _1→CVE_: 1.6; and TIR_2→CVE_: 3.4).

**Table 2:** Association of explanatory variables with depression symptom severity progression, regression, and cardiovascular event. Depression Level 1 = No Symptoms (<8), 2 = Mild (8–10), 3 = Moderate/Severe (≥11)^a^.

	Progression			Regression			*CVE*		
	1→2	1→3	2→3	2→1	3→1	3→2	*1→CVE*	*2→CVE*	*3→CVE*
Variables	TIR (95% CI)	TIR (95% CI)	TIR (95% CI)	TIR (95% CI)	TIR (95% CI)	TIR (95% CI)	*TIR* *(95% CI)*	*TIR* *(95% CI)*	*TIR* *(95% CI)*
Age	1.0 (0.8, 1.2)	1.1 (0.8,1.6)	1.1 (0.8, 1.5)	**0.8** **(0.6, 0.9)**	0.9 (0.6, 1.4)	0.7 (0.5, 1.1)	***1*.*4*** ***(1*.*2*, *1*.*8)***	***1*.*9*** ***(1*.*1*, *3*.*3)***	***2*.*3*** ***(1*.*3*, *3*.*9)***
Female Sex	1.0 (0.7, 1.5)	0.8 (0.4, 1.7)	0.8 (0.4, 1.7)	1.2 (0.8, 1.8)	1.4 (0.7, 2.8)	0.9 (0.4, 2.2)	***0*.*5*** ***(0*.*3*, *0*.*9)***	*1*.*0* *(0*.*3*, *1*.*5)*	*0*.*4* *(0*.*1*, *1*.*4)*
BMI	**1.3** **(1.0, 1.7)**	0.8 (0.6, 1.2)	1.1 (0.7, 1.5)	**0.7** **(0.6, 0.9)**	0.7 (0.5, 1.1)	1.2 (0.7, 1.8)	*0*.*9* *(0*.*7*, *1*.*2)*	*1*.*2* *(0*.*7*, *2*.*1)*	*1*.*4* *(0*.*8*, *2*.*6)*
Current/former smoker	0.9 (0.6, 1.2)	1.4 (0.8, 2.5)	1.1 (0.6, 1.9)	0.9 (0.6, 1.2)	0.7 (0.4, 1.3)	1.3 (0.6, 2.6)	*1*.*2* *(0*.*8*, *1*.*7)*	*1*.*8* *(0*.*7*, *4*.*7)*	*0*.*9* *(0*.*4*, *2*.*2)*
Education <10 years	**1.4** **(1.0, 1.8)**	**1.9** **(1.1, 3.3)**	1.1 (0.7, 1.9)	1.1 (0.8, 1.5)	1.8 (0.9, 3.6)	0.9 (0.5, 1.7)	*1*.*3* *(0*.*9*, *1*.*8)*	*1*.*1* *(0*.*5*, *2*.*5)*	***0*.*4*** ***(0*.*2*, *0*.*8)***
History of diabetes	1.1 (0.7, 1.6)	1.5 (0.8, 2.8)	1.3 (0.7, 2.4)	1.2 (0.8, 1.8)	1.1 (0.6, 2.1)	1.0 (0.5, 2.2)	***1*.*6*** ***(1*.*1*, *2*.*4)***	***3*.*4*** ***(1*.*4*, *7*.*8)***	*0*.*4* *(0*.*1*, *1*.*1)*
History of myocardial infarction	1.0 (0.8, 1.4)	1.4 (0.8, 2.4)	0.9 (0.5, 1.4)	1.2 (0.9, 1.6)	**0.6** **(0.3, 1.0)**	0.9 (0.5, 1.5)	*1*.*2* *(0*.*9*, *1*.*7)*	*1*.*3* *(0*.*6*, *2*.*8)*	*0*.*8* *(0*.*4*, *2*.*0)*
Lower physical activity level	**1.3** **(1.1, 1.5)**	**1.6** **(1.3, 2.1)**	1.2 (0.9, 1.5)	**0.8** **(0.7, 1.0)**	0.8 (0.6, 1.0)	**0.7** **(0.5, 1.0)**	*1*.*1* *(0*.*9*, *1*.*3)*	*1*.*4* *(0*.*9*, *1*.*9)*	*1*.*5* *(1*.*0*, *2*.*2)*
Anti-depression meds	0.9 (0.3, 2.9)	**3.6** **(1.3, 10.2)**	1.6 (0.4, 5.8)	0.9 (0.3, 3.1)	0.6 (0.1, 2.6)	0.3 (0.1, 1.8)	*0*.*0* *(0*.*0*, *0*.*0)*	*2*.*0* *(0*.*2*, *19*.*4)*	*3*.*2* *(0*.*6*, *16*.*8)*
Anti-anxiety meds	0.8 (0.2, 3.1)	1.6 (0.4, 6.8)	1.4 (0.4, 4.8)	1.2 (0.5, 3.0)	1.9 (0.3, 10.8)	3.5 (0.6, 19.3)	*1*.*9* *(0*.*7*, *5*.*3)*	*0*.*0* *(0*.*0*, *0*.*0)*	*0*.*0* *(0*.*0*, *0*.*0)*

Depression level 1 = no symptoms (<8), 2 = mild (8–10), 3 = moderate/severe (≥11); TIR, Transition Intensities Ratio; 95% CI, 95% Confidence Interval; CVE, cardiovascular event; BMI, body mass index.

^a^TIR estimates are adjusted for age (< 55 yr, 55–64 yr, and > 64 yr), female sex, body mass index (BMI; <25 kg/m^2^ normal, 25–29.9 kg/m^2^ overweight, and ≥30 kg/m^2^ obese), current/former smoker, education <10 years, history of diabetes, history of myocardial infarction, lower physical activity (PA) at year one (PA level 1: daily or 5-6x/week, PA level 2: 2-4x/week, PA level 3: 1x/week or 1-3x/month, and PA level 4: seldom or never), and anti-depression/anxiety medication use. TIR is the ratio of transition intensities between the exposed group to the unexposed group and can be interpreted as relative risk (RR).

Factors associated with the progression of anxiety were female sex (TIR_1→ 2_: 1.6) and lower physical activity groups (TIR_1→ 3_: 1.6; Table 3). History of a myocardial infarction was associated with a lower risk of regression (TIR_3→ 2_: 0.6). Similar to depression, older age groups were associated with a higher risk of a CVE and the association was stronger with increasing anxiety levels. Other factors associated with a CVE by anxiety levels included the following: female sex (TIR_1→CVE_: 0.5; TIR_3→CVE_: 0.1), history of diabetes (TIR_1→CVE_: 1.6), history of a myocardial infarction (TIR_3→CVE_: 4.2), lower physical activity groups (TIR_1→CVE_: 1.2; TIR_2→CVE_: 1.4), anti-depression medication use (TIR_3→CVE_: 16.7), and anti-anxiety medication use (TIR_1→CVE_: 3.0).

**Table 3:** Association of explanatory variables with anxiety symptom severity progression, regression, and cardiovascular event anxiety level 1 = no symptoms (<8), 2 = mild (8–10), 3 = moderate/severe (≥11)^a^.

	Progression			Regression			*CVE*		
	1→2	1→3	2→3	2→1	3→1	3→2	*1→CVE*	*2→CVE*	*3→CVE*
Variables	TIR (95% CI)	TIR (95% CI)	TIR (95% CI)	TIR (95% CI)	TIR (95% CI)	TIR (95% CI)	*TIR* *(95% CI)*	*TIR* *(95% CI)*	*TIR* *(95% CI)*
Age	**0.8** **(0.7, 1.0)**	0.8 (0.5, 1.1)	1.2 (0.9, 1.6)	0.9 (0.8, 1.1)	1.0 (0.7, 1.5)	1.2 (0.9, 1.6)	***1*.*5*** ***(1*.*2*, *1*.*9)***	***2*.*0*** ***(1*.*2*, *3*.*3)***	***2*.*2*** ***(1*.*1*, *4*.*2)***
Female Sex	**1.6** **(1.1, 2.2)**	1.6 (0.9, 3.1)	0.8 (0.4, 1.5)	1.1 (0.8, 1.5)	1.6 (0.8, 3.1)	1.0 (0.5, 1.8)	***0*.*5*** ***(0*.*3*, *0*.*9)***	*1*.*1* *(0*.*5*, *1*.*6)*	***0*.*1*** ***(0*.*02*, *0*.*5)***
BMI	1.1 (0.9, 1.4)	1.1 (0.7, 1.6)	0.9 (0.6, 1.3)	0.9 (0.7, 1.1)	0.9 (0.6, 1.3)	1.0 (0.7, 1.4)	*1*.*0* *(0*.*8*, *1*.*3)*	*0*.*9* *(0*.*5*, *1*.*5)*	*1*.*7* *(0*.*9*, *3*.*5)*
Current/ former smoker	0.9 (0.6, 1.1)	1.4 (0.8, 2.4)	1.1 (0.7, 1.8)	0.9 (0.6, 1.2)	1.1 (0.6, 2.1)	1.6 (0.9, 2.7)	*1*.*1* *(0*.*8*, *1*.*6)*	*1*.*7* *(0*.*8*, *3*.*8)*	*0*.*5* *(0*.*2*, *1*.*3)*
Education <10 years	1.1 (0.8, 1.4)	1.4 (0.8, 2.4)	1.0 (0.6, 1.6)	1.0 (0.8, 1.3)	0.8 (0.4, 1.4)	0.7 (0.5, 1.1)	*1*.*2* *(0*.*9*, *1*.*7)*	*0*.*5* *(0*.*3*, *1*.*1)*	*0*.*9* *(0*.*3*, *2*.*7)*
History of diabetes	0.7 (0.5, 1.1)	1.4 (0.7, 2.5)	0.6 (0.3, 1.2)	1.1 (0.8, 1.6)	0.9 (0.4, 2.2)	1.2 (0.6, 2.2)	***1*.*6*** ***(1*.*1*, *2*.*3)***	*1*.*9* *(0*.*8*, *4*.*5)*	*1*.*1* *(0*.*3*, *4*.*3)*
History of myocardial infarction	1.0 (0.8, 1.3)	0.9 (0.5, 1.4)	1.2 (0.8, 1.9)	1.1 (0.8, 1.4)	0.9 (0.5, 1.6)	**0.6** **(0.4, 0.9)**	*1*.*2* *(0*.*9*, *1*.*6)*	*1*.*3* *(0*.*6*, *2*.*6)*	***4*.*2*** ***(1*.*1*, *15*.*8)***
Lower physical activity level	1.1 (1.0, 1.3)	**1.6** **(1.3, 2.1)**	1.1 (0.9, 1.4)	1.0 (0.8, 1.1)	0.8 (0.6, 1.1)	0.9 (0.7, 1.1)	***1*.*2*** ***(1*.*0*, *1*.*4)***	***1*.*4*** ***(1*.*0*, *2*.*0)***	*1*.*5* *(0*.*8*, *2*.*7)*
Anti-depression meds	**2.2** **(1.0, 4.7)**	1.2 (0.2, 9.0)	1.2 (0.4, 3.4)	0.8 (0.4, 1.7)	0.6 (0.1, 3.0)	0.4 (0.1, 1.4)	*0*.*0* *(0*.*0*, *0*.*0)*	*0*.*0* *(0*.*0*, *0*.*0)*	***16*.*7*** ***(3*.*2*, *88*.*1)***
Anti-anxiety meds	1.8 (0.8, 4.2)	1.0 (0.1, 7.7)	2.0 (0.7, 5.4)	0.8 (0.3, 2.1)	0.8 (0.1, 7.3)	2.3 (0.7, 7.3)	***3*.*0*** ***(1*.*1*, *8*.*1)***	*0*.*0* *(0*.*0*, *0*.*0)*	*0*.*0* *(0*.*0*, *0*.*0)*

Anxiety level 1 = no symptoms (<8), 2 = mild (8–10), 3 = moderate/severe (≥11); TIR, Transition Intensities Ratio; 95% CI, 95% Confidence Interval; CVE, cardiovascular event; BMI, body mass index.

^a^TIR estimates are adjusted for age (< 55 yr, 55–64 yr, and > 64 yr), female sex, body mass index (BMI; <25 kg/m^2^ normal, 25–29.9kg/m^2^ overweight, and ≥30 kg/m^2^ obese), current/former smoker, education <10 years, history of diabetes, history of myocardial infarc tion, lower physical activity (PA) at year one (PA level 1: daily or 5-6x/week, PA level 2: 2-4x/week, PA level 3: 1x/week or 1-3x/month, and PA level 4: seldom or never), and anti-depression/anxiety medication use. TIR is the ratio of transition intensities between the exposed group to the unexposed group and can be interpreted as relative risk (RR).

## Discussion

In this cohort of participants with CHD and long-term follow-up, only about half of the participants with moderate or severe symptom severity at baseline transitioned to no symptom severity at 5 years, whereas at 10 years, the transition to no symptom severity ranged from 58–79% in younger participants to 47–58% in older participants. In addition, having both a higher symptom severity and lower physical activity were associated with a higher transition to a CVE at 5 and 10 years. Higher BMI, lower education, and lower physical activity were associated with progression of depression symptoms; lower physical activity and female sex were associated with progression of anxiety symptoms. Therefore, secondary prevention strategies of CHD should incorporate screening and treatment of depression and anxiety symptoms in order to improve long-term prognosis.

Our results are in agreement with previous studies showing a high burden of depression and anxiety among individuals with CHD [2–4, 18]. Following a cardiovascular event, the incidence of depression and anxiety is high in both the short [19] and long-term [20, 21] and its persistence is associated with considerable health care costs [22]. European [9, 23] and U.S. [7, 24] recommendations for secondary prevention of cardiovascular disease include screening and treating mood disorders. Still, depression and anxiety remain underdiagnosed [20] and undertreated [13, 21] in patients after a cardiovascular event. In this study, a third of the participants with mild or greater symptom severity at baseline had probabilities of the same or worse symptom severity at 5 and 10 years. Symptoms persist and may have a negative association on prognosis [2, 4, 25–27].

The highest probability of a cardiovascular event in this study was among those with moderate/severe depression and anxiety symptoms at baseline. Our results are in line with several previous studies showing that depression after a myocardial infarction is associated with a 2.6 to 2.7-fold risk of cardiovascular mortality [2, 25]. Similarly, anxiety after a myocardial infarction is associated with a 1.7-fold risk of a cardiovascular event [4] and a 1.2 to 3-fold risk of cardiovascular mortality [4, 26]. These observations support the need to identify depression and anxiety and to characterize trajectories of symptom severity in long-term management.

Female sex, lower education, and lifestyle factors are associated with a higher prevalence of depression and anxiety after a cardiovascular event [19, 28], but factors associated with progression of symptoms remain unknown. We observed an association between lower education, female sex, diabetes, higher BMI, and low physical activity with progression of depression and anxiety symptoms. Low socioeconomic status (i.e. low education)[29–31], females [28, 32], and those with diabetes [28] have a high burden of depression and anxiety and could be important subgroups for targeted screening in long-term care.

Low physical activity and BMI are modifiable, established risk factors for many diseases and adverse health outcomes, especially in older adults [12, 33], and could be important targets for managing the severity of depression and anxiety symptoms. Increasing physical activity has been shown to reduce symptoms of depression and anxiety [34, 35]. However, those with CHD are not achieving recommended targets for physical activity and risk factor management [36]. In this study, those in the lower two physical activity groups had a higher probability of worse depression and anxiety symptom severity and CVE after 5 and 10 years regardless of their depression status at baseline. Health behaviors, particularly physical inactivity, could be mediators of the association between symptoms of depression and anxiety and prognosis in individuals with CHD [10, 13]. These results suggest that cardiac rehabilitation and long-term care should include interventions to increase physical activity.

There is an opportunity to improve prognosis in CHD patients by providing cardiac rehabilitation sessions individualized to focus on risk factor management. In a recent meta-analysis, psychological treatments reduced the odds for all-cause mortality [37]. Although, the authors did not include studies with physical activity interventions that may be more beneficial in reducing morbidity and mortality after a cardiovascular event. A comprehensive cardiac rehabilitation program including diet, physical activity, and stress management improved lifestyle habits (increased physical activity and reduced BMI) and reduced cardiovascular mortality, non-fatal myocardial infarction, and stroke by 33% at 3 years [38]. A meta-analysis of six types of interventions in CHD found that psychological interventions reduced anxiety scores and behavioral interventions reduced all-cause mortality and non-fatal myocardial infarction [39]. While cardiac rehabilitation programs with psychological and physical activity interventions are effective in reducing depression and anxiety symptoms and improving prognosis, the development and implementation of these interventions needs further evaluation.

This study has the following limitations to consider. The majority of the study participants were male, thus results primarily pertain to males and may not necessarily apply to females in case of potential interaction by sex. Since participants were enrolled at least three weeks after their initial event, the study population may represent a healthier group of CHD patients and could have different depression and anxiety symptom severity compared to those who do not attend cardiac rehabilitation. Additionally, cardiac rehabilitation is standard treatment of CHD in Germany, but not all patients participate in the program and this could lead to underrepresentation of severely ill patients with CHD. However, in Germany, over 80% of patients participate in cardiac rehabilitation after a myocardial infarction [40]. Although we evaluated symptoms of depression and anxiety symptoms by validated and standardized tools, not a clinical diagnosis, there is the potential for misclassification. In addition, we measured these symptoms over relatively wide intervals. Lastly, participants self-reported physical activity at one year, which may have potential for misclassification.

In individuals with CHD, there is an opportunity and an important need to improve mental as well as physical health outcomes given the long-term burden of depression and anxiety symptoms. Depression, anxiety, and physical activity have important roles in prognosis following a cardiovascular event. Since the high and consistent burden of depression and anxiety symptoms in patients with CHD, these disorders should be consistently targeted in secondary prevention strategies including a physical activity component.

## Supporting information

S1 File (Fig A). Transitions in (A) depression and (B) anxiety symptom severity over time from baseline level and transitions to a cardiovascular event (CVE) at 10 years by age group with adjustments for gender, body mass index, smoking, education, diabetes, myocardial infarction, physical activity and anti-depression/anxiety medication; transition probabilities are shown at the reference level, N = 997. (Fig B). Transitions in (A) depression and (B) anxiety symptom severity over time from baseline level and transitions to a cardiovascular event (CVE) at 10 years by physical activity (PA) group with adjustments for gender, body mass index, smoking, education, diabetes, myocardial infarction, physical activity and anti-depression/anxiety medication; transition probabilities are shown at the reference level, N = 997.
(PDF)

S2 File (Table A). Transitions between depression symptoms at baseline and depression symptoms or a cardiovascular event at 5 and 10 years by age group with adjustments for gender, body mass index, smoking, education, diabetes, myocardial infarction, physical activity and anti-depression/anxiety medication; transition probabilities are shown at the reference level, N = 997. (Table B). Transitions between severity of anxiety symptoms at baseline and anxiety symptoms or a cardiovascular event at 5 and 10 years by age group with adjustments for gender, body mass index, smoking, education, diabetes, myocardial infarction, physical activity and anti-depression/anxiety medication; transition probabilities are shown at the reference level, N = 997. (Table C). Transitions between depression symptoms at baseline or a cardiovascular event at 5 and 10 years by physical activity level at one year with adjustments for gender, body mass index, smoking, education, diabetes, myocardial infarction, physical activity and anti-depression/anxiety medication; transition probabilities are shown at the reference level, N = 997. (Table D). Transitions between anxiety symptoms at baseline or a cardiovascular event at 5 and 10 years by physical activity level at one year by age group with adjustments for gender, body mass index, smoking, education, diabetes, myocardial infarction, and anti-depression/anxiety medication; transition probabilities are shown at the reference level, N = 997.
(PDF)

S3 File Baseline patient questionnaire. (PDF)
