# Supplementary material for: Multi-state models of transitions in depression and anxiety symptom severity and cardiovascular events in patients with coronary heart disease
Source: PLoS One. 2019 Mar 7;14(3):e0213334. doi: 10.1371/journal.pone.0213334 (PMC6405099; doi:10.1371/journal.pone.0213334)
Supplement: S1 File — (Fig A). Transitions in (A) depression and (B) anxiety symptom severity over time from baseline level and transitions to a cardiovascular event (CVE) at 10 years by age group with adjustments for gender, body mass index, smoking, education, diabetes, myocardial infarction, physical activity and anti-depression/anxiety medication; transition probabilities are shown at the reference level, N = 997. (Fig B). Transitions in (A) depression and (B) anxiety symptom severity over time from baseline level and transitions to a cardiovascular event (CVE) at 10 years by physical activity (PA) group with adjustments for gender, body mass index, smoking, education, diabetes, myocardial infarction, physical activity and anti-depression/anxiety medication; transition probabilities are shown at the reference level, N = 997. (PDF) [file pone.0213334.s001.pdf]

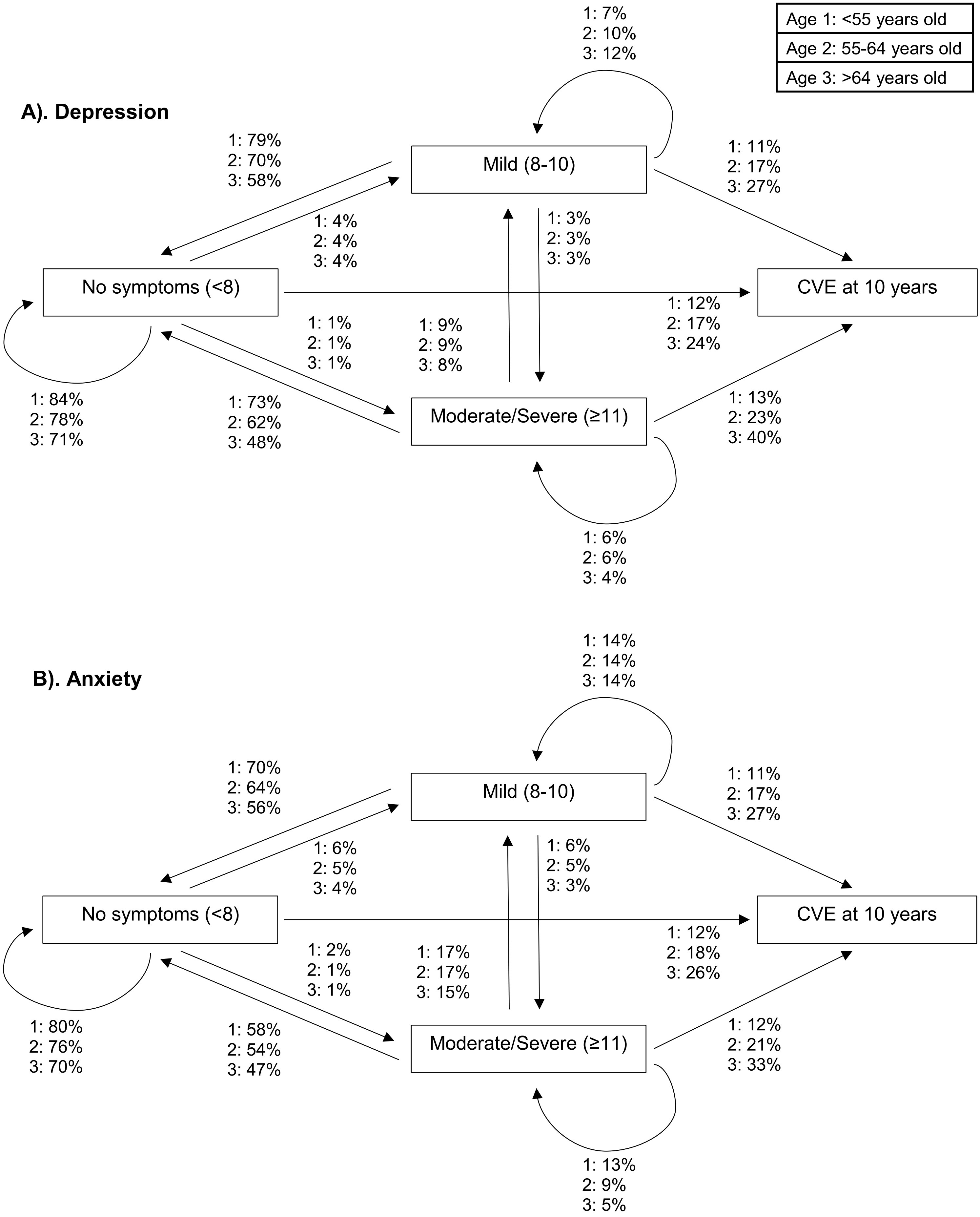

Figure A. Transitions in (A) depression and (B) anxiety symptom severity over time from baseline level and transitions to a cardiovascular event (CVE) at 10 years by age group with gender, BMI, smoking, education, diabetes, history of myocardial infarction, physical activity, and anti-depression/anxiety medication; transition probabilities are shown at the reference level, N = 997.

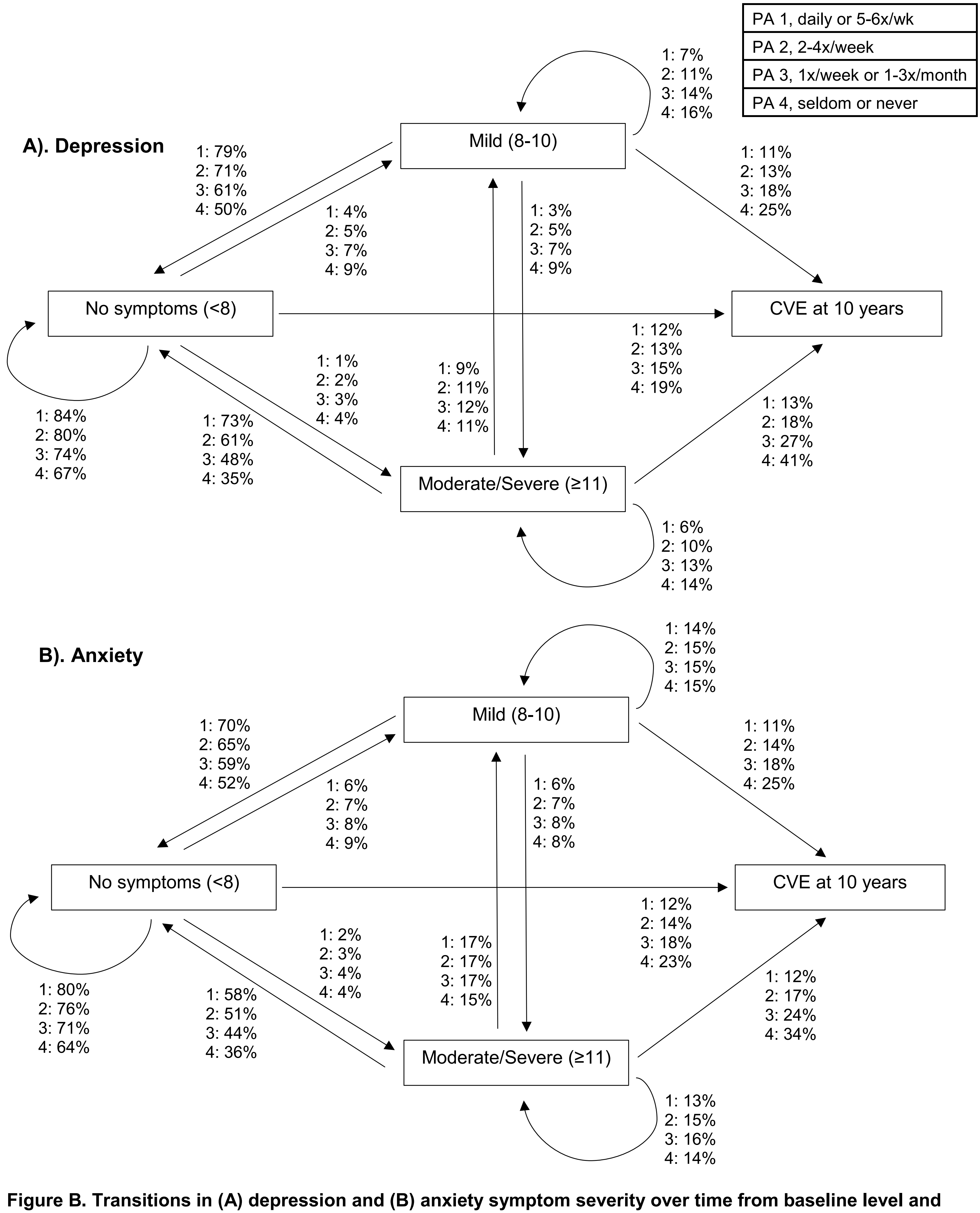

Figure B. Transitions in (A) depression and (B) anxiety symptom severity over time from baseline level and transitions to a cardiovascular event (CVE) at 10 years by physical activity (PA) group with gender, BMI, smoking, education, diabetes, history of myocardial infarction, physical activity, and anti-depression/anxiety medication; transition probabilities are shown at the reference level, N = 997.
