# Supplementary material for: Multi-state models of transitions in depression and anxiety symptom severity and cardiovascular events in patients with coronary heart disease
Source: PLoS One. 2019 Mar 7;14(3):e0213334. doi: 10.1371/journal.pone.0213334 (PMC6405099; doi:10.1371/journal.pone.0213334)
Supplement: S2 File — (Table A). Transitions between depression symptoms at baseline and depression symptoms or a cardiovascular event at 5 and 10 years by age group with adjustments for gender, body mass index, smoking, education, diabetes, myocardial infarction, physical activity and anti-depression/anxiety medication; transition probabilities are shown at the reference level, N = 997. (Table B). Transitions between severity of anxiety symptoms at baseline and anxiety symptoms or a cardiovascular event at 5 and 10 years by age group with adjustments for gender, body mass index, smoking, education, diabetes, myocardial infarction, physical activity and anti-depression/anxiety medication; transition probabilities are shown at the reference level, N = 997. (Table C). Transitions between depression symptoms at baseline or a cardiovascular event at 5 and 10 years by physical activity level at one year with adjustments for gender, body mass index, smoking, education, diabetes, myocardial infarction, physical activity and anti-depression/anxiety medication; transition probabilities are shown at the reference level, N = 997. (Table D). Transitions between anxiety symptoms at baseline or a cardiovascular event at 5 and 10 years by physical activity level at one year by age group with adjustments for gender, body mass index, smoking, education, diabetes, myocardial infarction, and anti-depression/anxiety medication; transition probabilities are shown at the reference level, N = 997. (PDF) [file pone.0213334.s002.pdf]

Table A. Transitions between depression symptoms at baseline and depression symptoms or a cardiovascular event at 5 and 10 years by age group with gender, body mass index, smoking, education, diabetes, myocardial infarction, physical activity and anti-depression/anxiety medication; transition probabilities are shown at the reference level, N = 997.

Level of depression symptoms at baseline by age groups	N	Transition probabilities (%) at 5 years				Transition probabilities (%) at 10 years			
		No symptoms (<8)	Mild (8-10)	Moderate/severe (≥11)	*CVE*	No symptoms (<8)	Mild (8-10)	Moderate/severe (≥11)	*CVE*
**No symptoms (<8)**									
< 55 yr	203	89.2	5.3	0.8	*4.6*	83.7	3.8	1.0	*11.5*
55 – 64 yr	383	86.9	5.4	1.0	*6.7*	78.2	4.1	1.1	*16.5*
> 64 yr	241	83.9	5.3	0.9	*9.8*	71.2	4.3	0.8	*23.7*
**Mild (8-10)**									
< 55 yr	26	63.0	27.4	5.8	*3.8*	79.1	7.3	2.7	*10.8*
55 – 64 yr	58	53.4	33.1	7.7	*5.9*	69.7	10.1	3.4	*16.9*
> 64 yr	30	44.0	37.6	8.2	*10.3*	57.6	12.4	2.8	*27.3*
**Moderate/severe (≥11)**									
< 55 yr	21	48.4	24.3	22.4	*4.8*	72.8	8.6	5.7	*12.9*
55 – 64 yr	24	42.8	21.9	25.7	*9.6*	61.6	9.3	6.0	*23.1*
> 64 yr	11	37.7	18.5	24.0	*19.8*	48.0	8.2	3.5	*40.3*

CVE: cardiovascular event; shaded area show same level of symptoms at baseline and during follow-up

Table B. Transitions between severity of anxiety symptoms at baseline and anxiety symptoms or a cardiovascular event at 5 and 10 years by age group with gender, body mass index, smoking, education, diabetes, myocardial infarction, physical activity and anti-depression/anxiety medication; transition probabilities are shown at the reference level, N = 997.

Level of anxiety symptoms at baseline by age groups	N	Probabilities (%) at 5 years				Probabilities (%) at 10 years			
		No symptoms (<8)	Mild (8-10)	Moderate/severe (≥11)	CVE	No symptoms (<8)	Mild (8-10)	Moderate/severe (≥11)	CVE
**No symptoms (<8)**									
< 55 yr	173	86.5	7.2	1.7	4.6	80.0	6.4	1.9	11.8
55 – 64 yr	361	85.8	5.9	1.4	6.9	76.0	5.2	1.3	17.5
> 64 yr	231	83.9	4.7	1.0	10.4	69.8	3.9	0.7	25.6
**Mild (8-10)**									
< 55 yr	46	53.9	32.5	10.1	3.5	69.5	14.2	5.6	10.7
55 – 64 yr	75	50.1	33.4	10.7	5.8	63.9	14.3	4.7	17.1
> 64 yr	36	45.7	33.8	10.6	9.8	56.0	13.7	3.1	27.2
**Moderate/severe (≥11)**									
< 55 yr	31	32.9	22.8	40.3	4.1	58.0	17.1	12.6	12.3
55 – 64 yr	29	32.2	24.9	35.4	7.5	53.7	16.9	8.8	20.6
> 64 yr	15	30.8	26.5	29.0	13.8	46.8	15.3	4.6	33.3

CVE: cardiovascular event; shaded area show same level of symptoms at baseline and during follow-up

Table C. Transitions between depression symptoms at baseline or a cardiovascular event at 5 and 10 years by physical activity level at one year with gender, body mass index, smoking, education, diabetes, history of myocardial infarction, physical activity and anti-depression/anxiety medication; transition probabilities are shown at the reference level, N = 997.

Level of depression symptoms at baseline by physical activity level	N	Transition probabilities (%) at 5 years				Transition probabilities (%) at 10 years			
		No symptoms (<8)	Mild (8-10)	Moderate/ severe (≥11)	CVE	No symptoms (<8)	Mild (8-10)	Moderate/ severe (≥11)	CVE
**No symptoms (<8)**									
PA level 1, daily or 5-6x/week	276	89.2	5.3	0.8	4.6	83.7	3.8	1.0	11.5
PA level 2, 2-4x/week	351	86.4	6.9	1.5	5.2	79.7	5.3	1.9	13.1
PA level 3, 1x/week or 1-3x/month	134	82.7	8.8	2.6	5.9	74.3	7.2	3.1	15.4
PA level 4, seldom or never	63	77.9	11.0	4.1	7.1	67.2	9.1	4.4	19.3
**Mild (8-10)**									
PA level 1, daily or 5-6x/week	25	63.0	27.4	5.8	3.8	79.1	7.3	2.7	10.8
PA level 2, 2-4x/week	42	54.1	32.4	9.1	4.5	71.3	10.5	5.0	13.2
PA level 3, 1x/week or 1-3x/month	32	45.2	36.3	12.7	5.8	61.4	13.5	7.4	17.6
PA level 4, seldom or never	15	36.8	38.8	16.1	8.4	50.2	15.8	9.1	25.0
**Moderate/severe (≥11)**									
PA level 1, daily or 5-6x/week	9	48.4	24.3	22.4	4.8	72.8	8.6	5.7	12.9
PA level 2, 2-4x/week	24	38.4	23.3	31.4	7.0	61.1	10.8	9.8	18.2
PA level 3, 1x/week or 1-3x/month	14	29.5	20.7	39.0	10.7	47.9	11.6	13.1	27.4
PA level 4, seldom or never	9	22.1	17.5	43.7	16.7	34.8	10.8	13.7	40.6

CVE: cardiovascular event; PA: physical activity measured at one year; shaded area show same level of symptoms at baseline and during follow-up

Table D. Transitions between anxiety symptoms at baseline or a cardiovascular event at 5 and 10 years by physical activity level at one year by age group with gender, body mass index, smoking, education, diabetes, history of myocardial infarction, and anti-depression/anxiety medication; transition probabilities are shown at the reference level, N = 997.

Level of anxiety symptoms at baseline by physical activity level	N	Transition probabilities (%) at 5 years				Transition probabilities (%) at 10 years			
		No symptoms (<8)	Mild (8-10)	Moderate/ severe (≥11)	CVE	No symptoms (<8)	Mild (8-10)	Moderate/ severe (≥11)	CVE
**No symptoms (<8)**									
PA level 1, daily or 5-6x/week	243	86.5	7.2	1.7	4.6	80.0	6.4	1.9	11.8
PA level 2, 2-4x/week	323	83.8	8.2	2.5	5.5	75.8	7.2	2.6	14.4
PA level 3, 1x/week or 1-3x/month	127	80.3	9.3	3.7	6.7	70.7	8.0	3.5	17.8
PA level 4, seldom or never	69	76.0	10.4	5.3	8.3	64.3	8.8	4.2	22.7
**Mild (8-10)**									
PA level 1, daily or 5-6x/week	54	53.9	32.5	10.1	3.5	69.5	14.2	5.6	10.7
PA level 2, 2-4x/week	60	50.6	32.9	11.9	4.5	64.5	14.8	6.8	13.8
PA level 3, 1x/week or 1-3x/month	35	47.1	33.1	13.9	5.9	58.8	15.2	7.7	18.3
PA level 4, seldom or never	8	43.3	32.9	15.8	7.9	52.3	15.1	7.9	24.7
**Moderate/severe (≥11)**									
PA level 1, daily or 5-6x/week	13	32.9	22.8	40.3	4.1	58.0	17.1	12.6	12.3
PA level 2, 2-4x/week	34	28.2	21.6	44.5	5.8	51.0	17.2	14.6	17.2
PA level 3, 1x/week or 1-3x/month	18	23.8	20.2	47.6	8.3	43.6	16.6	15.5	24.3
PA level 4, seldom or never	10	19.9	18.6	49.3	12.3	35.9	15.3	14.4	34.4

CVE: cardiovascular event; PA: physical activity measured at one year; shaded area show same level of symptoms at baseline and during follow-up
