# Supplementary material for: Multi-state models of transitions in depression and anxiety symptom severity and cardiovascular events in patients with coronary heart disease
Source: PLoS One. 2019 Mar 7;14(3):e0213334. doi: 10.1371/journal.pone.0213334 (PMC6405099; doi:10.1371/journal.pone.0213334)
Supplement: S3 File — (PDF) [file pone.0213334.s003.pdf]

LANGZEITERFOLGE
DER
***KAR*DI*OL*OGISCHEN**
***A*NSCHLUSSHEILBEHANDLUNG**

Patientenfragebogen

- zu Beginn der Reha -

ID-Nummer: [ ]

Ausgabedatum: |__|__|.|__|__|.|__|__|__|__| (Tag.Monat.Jahr)

Rückgabe bis zum: |__|__|.|__|__|.|__|__|__|__|

Rückgabe am: |__|__|.|__|__|.|__|__|__|__|

Sehr geehrte Teilnehmerin, sehr geehrter Teilnehmer der ***KAROLA***-Studie!

Sie sind zur Rehabilitation in diese Klinik gekommen, damit Ihre Gesundheit soweit wie möglich wiederhergestellt werden kann. Dazu möchten wir mit der ***KAROLA***-Studie (Langzeiterfolge der ***KAR***di***OL***ogischen ***A***nschlußheilbehandlung) einen wesentlichen Beitrag leisten. Wie Ihnen bereits erklärt wurde, geht es in der Studie darum, zu untersuchen, wie kurzfristige Erfolge der stationären Rehabilitation den langfristigen Krankheitsverlauf und die damit verbundenen sozialen und beruflichen Konsequenzen beeinflussen. Um diese Untersuchung erfolgreich durchführen zu können, möchten wir Sie nun mit der Beantwortung des folgenden Fragebogens um Ihre aktive Mithilfe bitten. Alle Angaben werden selbstverständlich streng vertraulich behandelt und ausschließlich in pseudonymisierter Form ausgewertet.

Wir möchten Ihnen nun noch einige konkrete Hinweise zum Ausfüllen des Fragebogens geben:

Der Fragebogen ist in sechs Bereiche unterteilt, die z.B. Ihre Krankheit, Ihre Lebensgewohnheiten oder persönliche Angaben betreffen. **Bitte gehen Sie Frage für Frage sorgfältig durch und beantworten Sie alle Fragen vollständig.** Sind Sie sich bei der Beantwortung der einen oder anderen Frage nicht ganz sicher, so geben Sie bitte den aus Ihrer Sicht bestmöglichen Schätzwert an. Nur vollständig und gewissenhaft ausgefüllte Bögen sichern den Erfolg und die Qualität dieser Studie und liefern gültige Ergebnisse.

Falls Sie Probleme bei der Beantwortung haben, wenden Sie sich bitte an unsere Studienmitarbeiter im Hause. Auch wenn Sie sonst Fragen zu der Studie haben, stehen Ihnen unsere Mitarbeiter selbstverständlich zur Verfügung.

<u>So wird es gemacht:</u>

Bitte verwenden Sie nur einen blauen oder schwarzen Kugelschreiber!
Schreiben Sie nur in GROSSBUCHSTABEN und nicht über die Kästchen hinaus!

Beim Ausfüllen des Bogens machen Sie bitte in die vorgesehenen Kästchen jeweils ein Kreuz

z.B. Geschlecht: ☒ Männlich ☐ Weiblich

Wo es erforderlich ist, tragen Sie bitte Zahlen ein,

z.B. Geburtsjahr | 1 | 9 | 4 | 8 |

oder schreiben Sie einen Text in die dafür vorgesehene Zeile,

z.B. erlernter Beruf: .....M.A.U.R.E.R...

1. Ihre Person und Ihr derzeitiger Gesundheitszustand

**1.**	Ihr Geschlecht: ☐ männlich ☐ weiblich
**2.**	Welche Staatsangehörigkeit haben Sie?<br>☐ Deutsch<br>☐ Nicht Deutsch |__|__|__| tragen Sie hier bitte das Kfz-Kennzeichen Ihres Staates ein
**3.**	Wann sind Sie geboren? |__|__| . |__|__| . |__|__|__|__| In welchem Land sind Sie geboren? ....<br>***Falls Sie nicht in Deutschland geboren sind:***<br>Seit wann leben Sie in Deutschland? seit |__|__|__|__| (Jahresangabe)
**4.**	Wann sind Sie zuletzt vor Ihrem Eintritt in diese Reha-Klinik zur stationären Behandlung im Krankenhaus gewesen?<br>Von |__|__| . |__|__| . |__|__|__|__| bis |__|__| . |__|__| . |__|__|__|__| Tag Monat Jahr Tag Monat Jahr
**5.**	Was war der Grund dieses Krankenhausaufenthaltes?<br>(Es sind mehrere Antworten möglich)<br>☐ Herzrhythmusstörungen<br>☐ Herzinfarkt<br>☐ Operativer Eingriff am Herzen bzw. an den Herzkranzgefäßen<br>☐ Angina pectoris (Druck- bzw. Engegefühl im Brustraum)<br>☐ Sonstiges, nämlich: ....

**6.**	Welche Untersuchungen bzw. medizinischen Behandlungen wurden bei diesem Krankenhausaufenthalt durchgeführt?<br>(Es sind mehrere Antworten möglich)<br><br>☐ Koronarangiographie (Darstellung der Herzkranzgefäße mit Hilfe eines Kontrastmittels)<br>☐ Koronarangioplastie (Aufdehnung von Engstellen der Herzkranzgefäße)<br>☐ Stent-Implantation<br>☐ Bypass-Operation<br>☐ Sonstiges, nämlich: ....

**7.**	Waren Sie zwischen diesem Krankenhausaufenthalt und Ihrem jetzigen Reha-Aufenthalt zu Hause?<br><br>☐ ja<br>☐ nein

8. Beurteilen Sie bitte Ihren derzeitigen Gesundheitszustand anhand der folgenden Fragen:

	**gar nicht**	**sehr leicht**	**leicht**	**mäßig**	**stark**	**sehr stark**
Haben Sie Schmerzen im Kreuz, im Nacken oder im Rücken?	☐	☐	☐	☐	☐	☐
Haben Sie Gelenkbeschwerden in den Schultern, Armen oder Händen?	☐	☐	☐	☐	☐	☐
Haben Sie Gelenkbeschwerden in den Hüften, Beinen oder Füßen?	☐	☐	☐	☐	☐	☐
Haben Sie Schmerzen in der Brust oder Herzgegend?	☐	☐	☐	☐	☐	☐
Werden Sie kurzatmig, wenn Sie bergan oder schnell gehen?	☐	☐	☐	☐	☐	☐
Haben Sie ein Druck- oder Völlegefühl im Leib?	☐	☐	☐	☐	☐	☐
Gibt es bei Ihnen Stuhlgangprobleme?	☐	☐	☐	☐	☐	☐
Leiden Sie tags- oder nachtsüber unter Husten?	☐	☐	☐	☐	☐	☐
Leiden Sie unter Schlafstörungen?	☐	☐	☐	☐	☐	☐
Fühlen Sie sich erschöpft?	☐	☐	☐	☐	☐	☐
Fühlen Sie sich innerlich angespannt und nervös?	☐	☐	☐	☐	☐	☐
Fühlen Sie sich entmutigt und traurig?	☐	☐	☐	☐	☐	☐

9. Wie beurteilen Sie insgesamt Ihr derzeitiges Leistungsvermögen?

**sehr schlecht**	**schlecht**	**weniger gut**	**zufrieden-stellend**	**gut**	**sehr gut**
☐	☐	☐	☐	☐	☐

**10.**	Ihre derzeitigen alltäglichen Sorgen und Probleme:	**gar nicht**	**sehr leicht**	**leicht**	**mäßig**	**stark**	**sehr stark**
	Machen Sie sich Sorgen um Ihre Gesundheit?	☐	☐	☐	☐	☐	☐
	Falls berufstätig: Wie sehr sorgen Sie sich, aus gesundheitlichen Gründen Ihren Beruf aufgeben zu müssen?	☐	☐	☐	☐	☐	☐
	Falls berufstätig: Tragen Sie sich mit dem Gedanken, sich vorzeitig berenten zu lassen?	☐ ja		☐ nein			

**11.**	Ich erwarte von der bevorstehenden Reha...	**trifft voll zu**	**trifft eher zu**	**trifft teils zu, teils nicht**	**trifft eher nicht zu**	**trifft überhaupt nicht zu**
	Wiederherstellung meiner Gesundheit	☐	☐	☐	☐	☐
	Erholung	☐	☐	☐	☐	☐
	Linderung von Beschwerden	☐	☐	☐	☐	☐
	Aufklärung über meine Erkrankung und ihre Risiko-faktoren	☐	☐	☐	☐	☐
	Hilfe beim Umgang mit meiner Krankheit zu Hause	☐	☐	☐	☐	☐
	Falls berufstätig:					
	Verbesserung meiner Leistungsfähigkeit im Beruf	☐	☐	☐	☐	☐
	Ausübung meiner bish. berufl. Tätigkeit nach der Reha	☐	☐	☐	☐	☐
	Beratung über mögliche Probleme am Arbeitsplatz	☐	☐	☐	☐	☐
	Info über berufliche Umschulungsmöglichkeiten	☐	☐	☐	☐	☐

12.	Wie würden Sie Ihren Gesundheitszustand jetzt, **am Anfang der Reha**, beurteilen?

**sehr schlecht**	**schlecht**	**weniger gut**	**zufrieden-stellend**	**gut**	**sehr gut**
☐	☐	☐	☐	☐	☐

13.	Wie sollte Ihr Gesundheitszustand nach Ihren jetzigen Erwartungen **am Ende der Reha** sein?

**sehr schlecht**	**schlecht**	**weniger gut**	**zufrieden-stellend**	**gut**	**sehr gut**
☐	☐	☐	☐	☐	☐

2. Ihre medizinische Vorgeschichte

1. Hat jemals ein **Arzt** bei Ihnen eine der folgenden Erkrankungen festgestellt? Bitte machen Sie für jede der Erkrankungen (einschließlich Ihrer aktuellen Erkrankung) eine entsprechende Angabe. Falls eine Erkrankung **ärztlicherseits** festgestellt wurde, geben Sie bitte zusätzlich das Jahr an, in dem die Erkrankung erstmals diagnostiziert wurde.

	**ja**	**Jahr der erst-maligen Diagnose durch einen Arzt**	**nein**
Durchblutungsstörungen am Herzen, Angina pectoris	☐	|__|__|__|__|	☐
Herzinfarkt	☐	|__|__|__|__|	☐
Herzschwäche, Herzinsuffizienz	☐	|__|__|__|__|	☐
Durchblutungsstörungen am Gehirn	☐	|__|__|__|__|	☐
Schlaganfall	☐	|__|__|__|__|	☐
Krampfadern, Venenentzündung	☐	|__|__|__|__|	☐
Thrombose	☐	|__|__|__|__|	☐
Lungenembolie	☐	|__|__|__|__|	☐
Bluthochdruck	☐	|__|__|__|__|	☐
Zuckerkrankheit, Diabetes mellitus	☐	|__|__|__|__|	☐
Gicht, Harnsäureerhöhung	☐	|__|__|__|__|	☐
Erhöhtes Cholesterin, erhöhte Blutfette	☐	|__|__|__|__|	☐
Asthma	☐	|__|__|__|__|	☐
Lungentuberkulose	☐	|__|__|__|__|	☐
Gallenblasenentzündung oder Gallensteine	☐	|__|__|__|__|	☐
Magen- oder Zwölffingerdarmgeschwür	☐	|__|__|__|__|	☐

1. *Fortsetzung von Frage 1*

	ja	Jahr der erstmaligen Diagnose durch einen Arzt	nein
Entzündung oder Steine der Blase, Niere oder Harnwege	☐	|__|__|__|__|	☐
Heuschnupfen	☐	|__|__|__|__|	☐
Neurodermitis, atopisches Ekzem	☐	|__|__|__|__|	☐
Andere Allergien	☐	|__|__|__|__|	☐
Krebserkrankungen	☐	|__|__|__|__|	☐

Krebserkrankungen: Falls ja, welche?

....

(Bitte möglichst ***genaue Angaben***, z.B. Lungenkrebs, Magenkrebs)

2. Ist bei Ihnen jemals eine der folgenden Operationen oder einer der folgenden Eingriffe durchgeführt worden? Falls ja, in welchem Jahr?
(Falls mehrere solche Operationen durchgeführt wurden, geben Sie bitte das Jahr der erstmaligen Operation an).

	ja	erstmals im Jahr	nein
Bypass-Operation, Operation an den Herzkranzgefäßen	☐	|__|__|__|__|	☐
Erweiterung der Herzkranzgefäße im Rahmen einer Untersuchung der Herzkranzgefäße (Koronarangiographie)	☐	|__|__|__|__|	☐
Krampfadernoperation	☐	|__|__|__|__|	☐
Gallenblasenoperation	☐	|__|__|__|__|	☐
Blinddarmoperation	☐	|__|__|__|__|	☐
Mandeloperation	☐	|__|__|__|__|	☐
Schilddrüsenoperation	☐	|__|__|__|__|	☐
Magenoperation wegen Geschwürserkrankung, Ulcusleiden	☐	|__|__|__|__|	☐
Krebsoperation	☐	|__|__|__|__|	☐

Krebsoperation: Falls ja, welche?

....

3. Wurden Ihnen ärztlicherseits jemals folgende Medikamente verordnet?
Falls ja, in welchem Jahr erstmals?

	**ja**	**erstmals im Jahr**	**nein**
Blutdrucksenkende Mittel	☐	|__|__|__|__|	☐
Tabletten zur Senkung des Blutzuckerspiegels	☐	|__|__|__|__|	☐
Insulin-Spritzen	☐	|__|__|__|__|	☐
Mittel zur Senkung des Blutfettspiegels	☐	|__|__|__|__|	☐
Mittel zur Senkung des Harnsäurespiegels	☐	|__|__|__|__|	☐
Antibiotika zur Ausrottung (Eradikation) einer Infektion mit dem Magenkeim *Helicobacter pylori*	☐	|__|__|__|__|	☐

4. Bitte schätzen Sie einmal, wie häufig bei Ihnen in den letzten 5 Jahren eine Antibiotikabehandlung durchgeführt wurde.

☐ keine Behandlung

☐ 1 Behandlung

☐ 2-3 Behandlungen

☐ 4-10 Behandlungen

☐ mehr als 10 Behandlungen

3. Angaben zu Ihrer Familie

1. In welchem Jahr ist **Ihr Vater** geboren?

|__|__|__|__|

2. Lebt **Ihr Vater** noch? ☐ ja
☐ nein, er ist im Jahr |__|__|__|__|

an .... gestorben.

3. Welche der folgenden Erkrankungen wurden **bei Ihrem Vater** jemals **ärztlicherseits** festgestellt? (Bitte machen Sie für jede der Erkrankungen eine entsprechende Angabe)

	**ja**	**nein**	**nicht bekannt weiß ich nicht**
Herzinfarkt	☐	☐	☐
Bluthochdruck	☐	☐	☐
Schlaganfall	☐	☐	☐
Zuckerkrankheit, Diabetes mellitus	☐	☐	☐
Krampfadern, Venenentzündung	☐	☐	☐
Thrombose	☐	☐	☐
Schwere geistige Verwirrtheit, z.B. Alzheimersche Erkrankung	☐	☐	☐
Asthma	☐	☐	☐
Magen- oder Zwölffingerdarmgeschwür	☐	☐	☐
Krebserkrankung	☐	☐	☐

Krebserkrankung: Falls ja, welche?

....

(Bitte möglichst ***genaue Angaben***, z.B. Lungen-, Magen-, Darm-, oder Nierenkrebs)

**4.**	In welchem Jahr ist **Ihre Mutter** geboren? |__|__|__|__|
**5.**	Lebt **Ihre Mutter** noch? ☐ ja<br>☐ nein, sie ist im Jahr |__|__|__|__|,<br>an .... gestorben
**6.**	Welche der folgenden Erkrankungen wurden **bei Ihrer Mutter** jemals **ärztlicherseits** festgestellt? (Bitte machen Sie für jede der Erkrankungen eine entsprechende Angabe)

	**ja**	**nein**	**nicht bekannt weiß ich nicht**
Herzinfarkt	☐	☐	☐
Bluthochdruck	☐	☐	☐
Schlaganfall	☐	☐	☐
Zuckerkrankheit, Diabetes mellitus	☐	☐	☐
Krampfadern, Venenentzündung	☐	☐	☐
Thrombose	☐	☐	☐
Schwere geistige Verwirrtheit, z.B. Alzheimersche Erkrankung	☐	☐	☐
Asthma	☐	☐	☐
Magen- oder Zwölffingerdarmgeschwür	☐	☐	☐
Krebserkrankung	☐	☐	☐

Krebserkrankung: Falls ja, welche?

....

(Bitte möglichst ***genaue Angaben***, z.B. Lungen-, Magen-, Darm-, oder Brustkrebs)

7. Welchen **höchsten** Schulabschluss haben/hatten Ihre Eltern?

Ihr Vater:

- ☐ Hauptschule/Volksschule
- ☐ Realschule/Mittlere Reife
- ☐ Polytechnische Oberschule
- ☐ Fachhochschulreife
- ☐ Abitur / allg. Hochschulreife
- ☐ anderen Schulabschluss

welchen:

....

- ☐ keinen Schulabschluss

Ihre Mutter:

- ☐ Hauptschule/Volksschule
- ☐ Realschule/Mittlere Reife
- ☐ Polytechnische Oberschule
- ☐ Fachhochschulreife
- ☐ Abitur / allg. Hochschulreife
- ☐ anderen Schulabschluss

....

- ☐ keinen Schulabschluss

8. Wie viele leibliche Geschwister haben oder hatten Sie?

Ich habe/hatte Geschwister |__|__| (Bitte Anzahl eintragen)

☐ Ich habe keine Geschwister

*In diesem Fall machen Sie bitte weiter mit **Abschnitt 4**.*

9. Wurde bei einem Ihrer Geschwister jemals **ärztlicherseits** eine der folgenden Erkrankungen festgestellt? Falls ja, geben Sie bitte an, bei wie vielen Ihrer Geschwister diese Erkrankung festgestellt wurde.

	**ja**	**festgestellt bei: Anzahl der Geschwister**	**nein**	**nicht bekannt weiß ich nicht**
Herzinfarkt	☐	|__|__|	☐	☐
Schlaganfall	☐	|__|__|	☐	☐
Krebserkrankung	☐	|__|__|	☐	☐

10. Bitte geben Sie für jedes Ihrer Geschwister das Geburtsjahr und das Geschlecht an. Bitte geben Sie auch an, ob Ihre Geschwister bereits verstorben sind und wenn ja, in welchem Jahr und woran.

Bitte füllen Sie für jedes Ihrer Geschwister eine Zeile aus.

Geburtsjahr / Geschlecht	männlich	weiblich	Bereits verstorben?	Wenn ja, Todesjahr:	Woran verstorben Todesursache:
1. |__|__|__|__|	☐	☐	☐nein ☐ja	|__|__|__|__|	....
2. |__|__|__|__|	☐	☐	☐nein ☐ja	|__|__|__|__|	....
3. |__|__|__|__|	☐	☐	☐nein ☐ja	|__|__|__|__|	....
4. |__|__|__|__|	☐	☐	☐nein ☐ja	|__|__|__|__|	....
5. |__|__|__|__|	☐	☐	☐nein ☐ja	|__|__|__|__|	....
6. |__|__|__|__|	☐	☐	☐nein ☐ja	|__|__|__|__|	....
7. |__|__|__|__|	☐	☐	☐nein ☐ja	|__|__|__|__|	....
8. |__|__|__|__|	☐	☐	☐nein ☐ja	|__|__|__|__|	....
9. |__|__|__|__|	☐	☐	☐nein ☐ja	|__|__|__|__|	....
10. |__|__|__|__|	☐	☐	☐nein ☐ja	|__|__|__|__|	....
11. |__|__|__|__|	☐	☐	☐nein ☐ja	|__|__|__|__|	....
12. |__|__|__|__|	☐	☐	☐nein ☐ja	|__|__|__|__|	....
13. |__|__|__|__|	☐	☐	☐nein ☐ja	|__|__|__|__|	....
14. |__|__|__|__|	☐	☐	☐nein ☐ja	|__|__|__|__|	....

4. Ihre Ausbildung und Berufstätigkeit

1. Welchen **höchsten** Schulabschluss haben Sie?

- ☐ Hauptschule/Volksschule
- ☐ Realschule/Mittlere Reife
- ☐ Polytechnische Oberschule
- ☐ Fachhochschulreife
- ☐ Abitur / allgemeine Hochschulreife
- ☐ anderer Schulabschluss:

....

- ☐ keinen Schulabschluss

2. Haben Sie eine abgeschlossene Berufsausbildung? (Bei mehreren Ausbildungen bitte die **zuletzt** abgeschlossene Ausbildung eintragen)

- ☐ gewerbliche oder landwirtschaftliche Lehre
- ☐ kaufmännische oder sonstige Lehre
- ☐ Berufsfachschule, Handelsschule
- ☐ Fachschule (z.B. Meister, Techniker)
- ☐ Beamtenausbildung
- ☐ Fachhochschule, Ingenieurschule
- ☐ Universität, Hochschule
- ☐ sonstiger Ausbildungsabschluss: ....
- ☐ keine abgeschlossene Ausbildung

**3.**	Waren Sie zuletzt (d.h. bevor Sie im Akutkrankenhaus gewesen sind) berufstätig?<br>☐ ja, voll berufstätig<br>☐ ja, in Teilzeit berufstätig, mindestens halbtags<br>☐ ja, stundenweise berufstätig<br>☐ nein, arbeitslos, seit: |__|__|.|__|__|__|__| (Monat/Jahr)<br>☐ nein, Hausfrau/Hausmann<br>☐ nein, in Ausbildung<br>☐ nein, altershalber in Rente/pensioniert, seit: |__|__|.|__|__|__|__| (Monat/Jahr)<br>☐ nein, vorzeitig aus gesundheitlichen Gründen in Rente,<br>seit: |__|__|.|__|__|__|__| (Monat/Jahr)<br>☐ nein, vorzeitig aus sonstigen Gründen in Rente,<br>seit: |__|__|.|__|__|__|__| (Monat/Jahr)

**4.**	Wann - in welchem Jahr - waren Sie zum ersten Mal hauptberuflich (mindestens halbtags) erwerbstätig?<br>|__|__|__|__| (Jahr)<br>☐ bin nie erwerbstätig gewesen<br>*In diesem Fall machen Sie bitte weiter mit **Frage 11***

**5.**	Wie viele Jahre sind/waren Sie *insgesamt* hauptberuflich (mindestens halbtags) erwerbstätig?<br>|__|__| Jahre

**6.**	Welche berufliche Tätigkeit üben Sie zur Zeit aus bzw. (falls nicht mehr berufstätig) haben Sie zuletzt ausgeübt? Machen Sie bitte möglichst genaue Angaben (z.B. *CHEMIEARBEITER IM SCHICHTBETRIEB*)<br>..

7.	Entspricht Ihre derzeitige bzw. letzte berufliche Tätigkeit Ihrem ursprünglich erlernten Beruf?<br>☐ ja<br>☐ nein, mein erlernter Beruf ist: ....<br>☐ ich habe keinen Beruf erlernt

8.	Welche hauptberufliche Tätigkeit haben Sie am längsten ausgeübt?<br>☐ die derzeitige, zuletzt ausgeübte<br>☐ meinen ursprünglich erlernten Beruf<br>☐ eine andere Tätigkeit, nämlich: ....<br><br>Wie lange haben Sie diese Tätigkeit hauptberuflich (mindestens halbtags) ausgeübt? |__|__| Jahre

9.	In welcher beruflichen Stellung sind Sie derzeit bzw. (falls nicht mehr berufstätig) waren Sie zuletzt beschäftigt?<br>☐ Arbeiter<br>☐ Angestellter<br>☐ Beamter<br>☐ Selbstständig<br>☐ Sonstiges, nämlich: ....

10.	Waren Sie in den letzten 5 Jahren einmal arbeitslos?<br>☐ nein<br>☐ ja, und zwar insgesamt: |__|__| Jahre |__|__| Monate<br><br>Zuletzt arbeitslos von: |__|__|.|__|__|__|__| (Monat/Jahr)<br>bis: |__|__|.|__|__|__|__| (Monat/Jahr)

11. Wie würden Sie Ihre **körperliche Beanspruchung** durch Ihre **Haupttätigkeit** (berufliche Tätigkeit, Ausbildung oder Haushalt) in verschiedenen Lebensabschnitten charakterisieren?
Bitte kreuzen Sie für jede Altersstufe bis einschließlich Ihrer jetzigen Altersstufe die am ehesten zutreffende Kategorie an.

	**körperlich anstrengende Tätigkeit (z.B. im Baugewerbe,- in der Landwirtschaft)**	**körperlich leichte bis mäßig schwere Tätigkeit**	**körperlich nicht anstrengende Tätigkeit (z.B. überwiegend sitzende Tätigkeit**
15-19 Jahre	☐	☐	☐
20-29 Jahre	☐	☐	☐
30-39 Jahre	☐	☐	☐
40-49 Jahre	☐	☐	☐
50-59 Jahre	☐	☐	☐
60-70 Jahre	☐	☐	☐
älter als 70 Jahre	☐	☐	☐

12. Wie würden Sie die **psychische, seelische oder nervliche Belastung (Streß)** durch Ihre **Haupttätigkeit** (berufliche Tätigkeit, Ausbildung oder Haushalt) in verschiedenen Lebensabschnitten beurteilen?
Bitte kreuzen Sie für jede Altersstufe bis einschließlich Ihrer jetzigen Altersstufe die am ehesten zutreffende Kategorie an.

	**starke psychische Belastung (z.B. hoher Zeit- und Leistungsdruck)**	**mäßige psychische Belastung**	**geringe psychische Belastung**
15-19 Jahre	☐	☐	☐
20-29 Jahre	☐	☐	☐
30-39 Jahre	☐	☐	☐
40-49 Jahre	☐	☐	☐
50-59 Jahre	☐	☐	☐
60-70 Jahre	☐	☐	☐
älter als 70 Jahre	☐	☐	☐

13. Wie würden Sie die **Zufriedenheit** mit Ihrer **Haupttätigkeit** (berufliche Tätigkeit, Ausbildung oder Haushalt) in verschiedenen Lebensabschnitten beurteilen?
Bitte kreuzen Sie für jede Altersstufe bis einschließlich Ihrer jetzigen Altersstufe die am ehesten zutreffende Kategorie an.

	**sehr zufrieden**	**eher zufrieden**	**eher unzufrieden**	**sehr unzufrieden**
15-19 Jahre	☐	☐	☐	☐
20-29 Jahre	☐	☐	☐	☐
30-39 Jahre	☐	☐	☐	☐
40-49 Jahre	☐	☐	☐	☐
50-59 Jahre	☐	☐	☐	☐
60-70 Jahre	☐	☐	☐	☐
älter als 70 Jahre	☐	☐	☐	☐

5. Ihre Lebensgewohnheiten

Körperliche Aktivität in der Freizeit

1.

In welchem Umfang haben Sie im Laufe Ihres Lebens während Ihrer Freizeit im Durchschnitt **körperlich anstrengende, schweißtreibende** Aktivitäten (z.B. Radfahren, zügiges Wandern, Gartenarbeit, Sport) ausgeübt?

Bitte kreuzen Sie für jede Altersstufe bis einschließlich Ihrer jetzigen Altersstufe die am ehesten zutreffende Kategorie an.

	**täglich**	**5-6 mal/ Woche**	**2-4 mal/ Woche**	**1 mal/ Woche**	**1-3 mal/ Monat**	**selten/nie**
15-19 Jahre	☐	☐	☐	☐	☐	☐
20-29 Jahre	☐	☐	☐	☐	☐	☐
30-39 Jahre	☐	☐	☐	☐	☐	☐
40-49 Jahre	☐	☐	☐	☐	☐	☐
50-59 Jahre	☐	☐	☐	☐	☐	☐
60-70 Jahre	☐	☐	☐	☐	☐	☐
älter als 70 Jahre	☐	☐	☐	☐	☐	☐

2.

In welchem Umfang haben Sie **innerhalb des letzten Jahres** während Ihrer Freizeit im Durchschnitt **körperlich anstrengende, schweißtreibende** Aktivitäten (z.B. Radfahren, zügiges Wandern, Gartenarbeit, Sport) ausgeübt?

Lassen Sie dabei Zeiten von Krankenhausaufenthalten unberücksichtigt.

Durchschnittlich |__|__| Stunden pro Woche.

Gewicht

3. Bitte versuchen Sie sich einmal zu erinnern, wie viel Sie während der angegebenen Altersspannen gewogen haben.

Bitte machen Sie Angaben zu Ihrem **geringsten** und **höchsten** Gewicht (ohne Schuhe) für jede Altersstufe bis einschließlich Ihrer jetzigen Altersstufe und runden Sie dabei auf ganze Zahlen. Sollte Ihr Gewicht nicht geschwankt haben, tragen Sie bitte in beide Spalten das gleiche Gewicht ein.

Für Frauen: Zeiten der Schwangerschaft sollten für die Gewichtsangaben unberücksichtigt bleiben.

	**geringstes Gewicht**	**höchstes Gewicht**
20-29 Jahre	|__|__|__| kg	|__|__|__| kg
30-39 Jahre	|__|__|__| kg	|__|__|__| kg
40-49 Jahre	|__|__|__| kg	|__|__|__| kg
50-59 Jahre	|__|__|__| kg	|__|__|__| kg
60-70 Jahre	|__|__|__| kg	|__|__|__| kg
älter als 70 Jahre	|__|__|__| kg	|__|__|__| kg

4. Wie häufig haben Sie seit Ihrem 20. Lebensjahr **willentlich (durch Diät)** um mehr als 5 kg innerhalb von weniger als 12 Monaten abgenommen?

Bitte geben Sie die bestmögliche Schätzung für jede Altersstufe einschließlich Ihrer jetzigen Altersstufe an, und zwar unabhängig davon, wie lange die Gewichtsabnahme angehalten hat. Haben Sie in einer Altersstufe keine Diät gemacht, tragen Sie bitte eine 0 ein.

20-29 Jahre	|__|__|__| mal
30-39 Jahre	|__|__|__| mal
40-49 Jahre	|__|__|__| mal
50-59 Jahre	|__|__|__| mal
60-70 Jahre	|__|__|__| mal
älter als 70 Jahre	|__|__|__| mal

**5.**	Wie viel haben Sie vor 12 Monaten gewogen (ohne Schuhe)?<br><br>|__|__|__| kg<br><br>Haben Sie innerhalb der letzten 12 Monate um mehr als 1 kg zugenommen oder abgenommen?<br><br>☐ zugenommen und zwar um |__|__| kg<br><br>☐ weder noch<br><br>☐ abgenommen und zwar um |__|__| kg<br><br>Wie groß sind Sie (ohne Schuhe)? |__|__|__| cm

Rauchen

Zigaretten

**6.**	Haben Sie in Ihrem Leben jemals **regelmäßig** Zigaretten geraucht? (mindestens eine Zigarette pro Tag)<br><br>☐ ja<br><br>☐ nein *In diesem Fall machen Sie bitte weiter mit **Frage 13***

**7.**	Wie alt waren Sie, als Sie begonnen haben, regelmäßig Zigaretten zu rauchen? |__|__| Jahre

**8.**	Haben Sie **aufgehört**, regelmäßig Zigaretten zu rauchen?<br><br>☐ ja<br><br>☐ nein *In diesem Fall machen Sie bitte weiter mit **Frage 12***

**9.**	Wann haben Sie zuletzt aufgehört, regelmäßig Zigaretten zu rauchen?<br><br>☐ innerhalb des letzten Monats<br><br>☐ vor 1-3 Monaten<br><br>☐ vor 4-6 Monaten<br><br>☐ vor 7-12 Monaten<br><br>☐ vor mehr als 12 Monaten, und zwar im Alter von |__|__| Jahren.

**10.**	Was war der **wichtigste** Grund für Sie, mit dem Rauchen aufzuhören? (Bitte nur eine, die am ehesten zutreffende Antwort ankreuzen)<br><br>☐ eine akut aufgetretene Erkrankung, nämlich: ....<br>☐ eine seit längerem (mindestens 12 Monate) bestehende, chronische Erkrankung, nämlich: ....<br>☐ der Wunsch, möglichen Erkrankungen durch das Zigarettenrauchen vorzubeugen<br>☐ das Drängen von Angehörigen, Bekannten oder Freunden<br>☐ die Zigaretten haben mir nicht mehr geschmeckt<br>☐ die finanziellen Kosten des Rauchens<br>☐ ein sonstiger Grund, nämlich ....
**11.**	Wie viele Zigaretten pro Tag haben Sie während der Zeit Ihres **regelmäßigen Rauchens** durchschnittlich geraucht?<br><br>|__|__| Zigaretten/Tag
**12.**	Wie viele Zigaretten haben Sie in den folgenden Zeiträumen durchschnittlich pro Tag geraucht?<br><br>In den letzten 4 Wochen: |__|__| Zigaretten/Tag<br>Innerhalb der letzten 12 Monate: |__|__| Zigaretten/Tag<br>Davor, d.h. während der übrigen Zeit Ihres regelmäßigen Rauchens:<br>|__|__| Zigaretten/Tag

Zigarren/Pfeife

**13.**	Haben Sie in Ihrem Leben jemals **regelmäßig** Zigarren oder Pfeife geraucht? (mindestens drei Zigarren oder Pfeifen pro Woche)<br><br>☐ ja<br><br>☐ nein *In diesem Fall machen Sie bitte weiter mit **Frage 16***

**14.**	Wie lange haben Sie insgesamt regelmäßig Zigarren oder Pfeife geraucht?<br><br>|__|__| Jahre |__|__| Monate

**15.**	Wie viele Zigarren oder Pfeifen haben Sie während dieser Zeit durchschnittlich pro Woche geraucht?<br><br>|__|__|__| Zigarren/Pfeifen pro Woche.

Alkohol

16. Bitte versuchen Sie sich einmal zu erinnern, welche Mengen der folgenden alkoholischen Getränke Sie **im Laufe Ihres bisherigen Lebens** im Durchschnitt ***pro Woche*** getrunken haben.

Bitte geben Sie für jede Altersstufe bis einschließlich Ihrer jetzigen Altersstufe die bestmögliche Schätzung an und runden Sie die Gläser bzw. Flaschen pro Woche dabei auf ganze Zahlen.

Wein, Sekt, Obstwein (Most)

15-19 Jahre	|__|__|__|	Gläser (1/4 l) pro Woche
20-29 Jahre	|__|__|__|	Gläser (1/4 l) pro Woche
30-39 Jahre	|__|__|__|	Gläser (1/4 l) pro Woche
40-49 Jahre	|__|__|__|	Gläser (1/4 l) pro Woche
50-59 Jahre	|__|__|__|	Gläser (1/4 l) pro Woche
60-70 Jahre	|__|__|__|	Gläser (1/4 l) pro Woche
älter als 70 Jahre	|__|__|__|	Gläser (1/4 l) pro Woche

Bier

15-19 Jahre	|__|__|__|	Flaschen (0,5 l) pro Woche
20-29 Jahre	|__|__|__|	Flaschen (0,5 l) pro Woche
30-39 Jahre	|__|__|__|	Flaschen (0,5 l) pro Woche
40-49 Jahre	|__|__|__|	Flaschen (0,5 l) pro Woche
50-59 Jahre	|__|__|__|	Flaschen (0,5 l) pro Woche
60-70 Jahre	|__|__|__|	Flaschen (0,5 l) pro Woche
älter als 70 Jahre	|__|__|__|	Flaschen (0,5 l) pro Woche

Hochprozentige alkoholische Getränke (Schnaps, Likör, Rum, Weinbrand...)

15-19 Jahre	|__|__|__|	Gläser (2 cl) pro Woche
20-29 Jahre	|__|__|__|	Gläser (2 cl) pro Woche
30-39 Jahre	|__|__|__|	Gläser (2 cl) pro Woche
40-49 Jahre	|__|__|__|	Gläser (2 cl) pro Woche
50-59 Jahre	|__|__|__|	Gläser (2 cl) pro Woche
60-70 Jahre	|__|__|__|	Gläser (2 cl) pro Woche
älter als 70 Jahre	|__|__|__|	Gläser (2 cl) pro Woche

**17.**	Welche Mengen der folgenden alkoholischen Getränke haben Sie **innerhalb der letzten 12 Monate** im Durchschnitt getrunken?<br>Bitte geben Sie für jeden Zeitraum die bestmögliche Schätzung an und runden Sie die Gläser bzw. Flaschen pro Woche dabei auf ganze Zahlen.<br><br>**Wein, Sekt, Obstwein (Most)**<br>Innerhalb der letzten Woche |__|__|__| Gläser (1/4 l)<br>Innerhalb der letzten 4 Wochen |__|__|__| Gläser (1/4 l) ***pro Woche***<br>Innerhalb der letzten 12 Monate |__|__|__| Gläser (1/4 l) ***pro Woche***<br><br>**Bier**<br>Innerhalb der letzten Woche |__|__|__| Flaschen (0,5 l)<br>Innerhalb der letzten 4 Wochen |__|__|__| Flaschen (0,5 l) ***pro Woche***<br>Innerhalb der letzten 12 Monate |__|__|__| Flaschen (0,5 l) ***pro Woche***<br><br>**Hochprozentige alkoholische Getränke (Schnaps, Likör, Rum, Weinbrand, u.ä.)**<br>Innerhalb der letzten Woche |__|__|__| Gläser (2 cl)<br>Innerhalb der letzten 4 Wochen |__|__|__| Gläser (2 cl) ***pro Woche***<br>Innerhalb der letzten 12 Monate |__|__|__| Gläser (2 cl) ***pro Woche***

Ernährung

18. Wie oft haben Sie die folgenden Nahrungsmittel **in den letzten 12 Monaten** vor Ihrer stationären Aufnahme im Akutkrankenhaus zu sich genommen? Denken Sie bitte bei der Beantwortung dieser Frage an alle Mahlzeiten, also an **Frühstück, Mittagessen, Abendessen** und an alle **Zwischenmahlzeiten**.

Bitte geben Sie für jede Lebensmittelgruppe an, wie viele Portionen Sie im Durchschnitt davon verzehrt haben und in welchem Zeitraum Sie die angegebene Anzahl Portionen verzehrt haben.

Falls sich Ihre Ernährungsgewohnheiten innerhalb dieses Jahres geändert haben, so bilden Sie bitte den Durchschnitt über das ganze Jahr.

Im folgenden zeigen wir Ihnen in 3 möglichen Beispielen, wie Sie die Eintragungen vornehmen sollten:

Beispiel 1: Sie haben im Durchschnitt morgens 2 Scheiben Brot und abends 2 Scheiben Brot, also insgesamt 4 Scheiben am Tag gegessen. Dann tragen Sie bitte unter **Anzahl der Portionen** *4* ein und kreuzen **pro Tag** an (siehe unten).

Beispiel 2: Sie haben im Durchschnitt 2 mal in der Woche Reis, einmal 1 Portion und einmal 2 Portionen, also insgesamt 3 Portionen in der Woche gegessen. Dann tragen Sie bitte unter **Anzahl der Portionen** *3* ein und kreuzen **pro Woche** an (siehe unten).

Beispiel 3: Sie haben ungefähr 3 mal im Monat jeweils 2 Gläser Wein, also insgesamt 6 Gläser Wein im Monat getrunken. Dann tragen Sie bitte unter **Anzahl der Portionen** *6* ein und kreuzen **pro Monat** an (siehe unten).

	**Anzahl der Portionen, Gläser, etc.**	**In welchem Zeitraum?** pro Tag	pro Woche	pro Monat	nie
Beispiele (1-3)					
1. Brot, Brötchen (1 Scheibe bzw. 1 Brötchen)	|__|3_|	☒	☐	☐	☐
2. Reis, Grünkern, Hirse, etc. (1 Portion)	|__|1__|	☐	☒	☐	☐
3. Wein, Sekt, Obstwein (Most) (1 Glas, ca. 0,25 l)	|__|2__|	☐	☒	☐	☐
Obst und Gemüse					
Frisches Obst (1 Stück bzw. 1 Portion)	|__|__|	☐	☐	☐	☐
Salat oder Gemüse, roh (1 Portion)	|__|__|	☐	☐	☐	☐
Gemüse, gekocht (1 Portion)	|__|__|	☐	☐	☐	☐

	Fortsetzung Frage ***18***	**Anzahl der Portionen, Gläser, etc.**	**In welchem Zeitraum?** pro Tag	pro Woche	pro Monat	nie
	Fleisch und Wurstwaren					
	Fleisch inkl. Geflügel, ohne Wurstwaren (1 Portion, ca. 150 g)	|__|__|	☐	☐	☐	☐
	magere Wurst (z.B. Geflügelwurst, gekochter Schinken, etc.) (1 Scheibe bzw. 1 Portion)	|__|__|	☐	☐	☐	☐
	sonstige Wurst (1 Scheibe bzw. 1 Portion)	|__|__|	☐	☐	☐	☐
	Fisch (1 Portion, ca. 200 g)	|__|__|	☐	☐	☐	☐
	Beilagen					
	Salzkartoffeln, Knödel (1 Portion)	|__|__|	☐	☐	☐	☐
	Pommes frites, Kroketten, Bratkartoffeln (1 Portion)	|__|__|	☐	☐	☐	☐
	Nudeln, Spätzle (1 Portion)	|__|__|	☐	☐	☐	☐
	Reis, Grünkern, Hirse, etc. (1 Portion)	|__|__|	☐	☐	☐	☐
	Getreideerzeugnisse					
	Vollkornbrot, -brötchen (1 Scheibe bzw. 1 Brötchen)	|__|__|	☐	☐	☐	☐
	Brot, Brötchen (1 Scheibe bzw. 1 Brötchen)	|__|__|	☐	☐	☐	☐
	Haferflocken, Müsli, Cornflakes (1 Portion)	|__|__|	☐	☐	☐	☐

*Fortsetzung Frage **18***	**Anzahl der Portionen, Gläser, etc.**	**In welchem Zeitraum?** pro Tag	pro Woche	pro Monat	nie
Eier, Milch und Milchprodukte					
Eier (1 Ei)	|__|__|	☐	☐	☐	☐
Milch, Kakao (1 Glas, ca. 0,2 l)	|__|__|	☐	☐	☐	☐
Halbfettmargarine (1 Portion, ca. 10 g)	|__|__|	☐	☐	☐	☐
Butter, sonstige Margarine (1 Portion, ca. 10 g)	|__|__|	☐	☐	☐	☐
Quark, Joghurt, Dickmilch, Buttermilch (1 Becher, ca. 150 g)	|__|__|	☐	☐	☐	☐
geschlagene Sahne (1 Portion)	|__|__|	☐	☐	☐	☐
Käse mit reduziertem Fettgehalt (1 Scheibe bzw. 1 Portion)	|__|__|	☐	☐	☐	☐
sonstiger Käse (1 Scheibe bzw. 1 Portion)	|__|__|	☐	☐	☐	☐
Süßwaren					
Pudding, Kompott, Eis (1 Portion)	|__|__|	☐	☐	☐	☐
Marmelade, Honig (1 Teelöffel)	|__|__|	☐	☐	☐	☐
Zucker (1 Teelöffel)	|__|__|	☐	☐	☐	☐
Schokolade, Pralinen, Schokoriegel (1 Riegel bzw. vergleichbare Menge)	|__|__|	☐	☐	☐	☐
Kuchen, Gebäck, Kekse (1 Kuchenstück bzw. vergleichbare Menge)	|__|__|	☐	☐	☐	☐

*Fortsetzung Frage **18***	**Anzahl der Portionen, Gläser, etc.**	**In welchem Zeitraum?** pro Tag	pro Woche	pro Monat	nie
Salzige Esswaren					
salzige Knabbereien (z.B. Chips) (1 Portion, ca. 100 g)	|__|__|	☐	☐	☐	☐
Fertiggerichte (z.B. Suppen, Pizza, Nudel-gerichte, etc.) (1 Portion)	|__|__|	☐	☐	☐	☐
Alkoholfreie Getränke					
Mineralwasser (1 Glas, ca. 0,2 l)	|__|__|	☐	☐	☐	☐
Kalorienreduzierte Getränke (z.B. Cola light) (1 Glas, ca. 0,2 l)	|__|__|	☐	☐	☐	☐
sonstige Erfrischungs-getränke (z.B. Obstsaft, Cola) (1 Glas, ca. 0,2 l)	|__|__|	☐	☐	☐	☐
Kaffee, Tee					
Kaffee (1 Tasse)	|__|__|	☐	☐	☐	☐
Tee (1 Tasse)	|__|__|	☐	☐	☐	☐
Alkohol					
Bier (1 Flasche, 0,5 l)	|__|__|	☐	☐	☐	☐
Wein, Sekt, Obstwein (Most) (1 Glas, ca. 0,25 l)	|__|__|	☐	☐	☐	☐
Spirituosen (Schnaps, Likör, Weinbrand, u.ä.) (1 Glas, ca. 2 cl)	|__|__|	☐	☐	☐	☐

19.	Wenn Sie an die **letzten 12 Monate** vor Ihrer stationären Aufnahme im Akutkrankenhaus denken, wie sind/waren Sie es gewohnt, zu Hause Ihre Speisen zuzubereiten?<br>☐ eher deftig, mit viel Soße<br>☐ eher schonend im eigenen Saft gegart<br>☐ sonstiges, und zwar: ....

6. Ihr persönliches Umfeld

**1.**	Welchen Familienstand haben Sie?<br>☐ ledig<br>☐ verheiratet<br>☐ geschieden<br>☐ verwitwet

**2.**	Leben Sie mit einem Partner/einer Partnerin zusammen?<br>☐ ja<br>☐ nein *In diesem Fall machen Sie bitte weiter mit **Frage 4***

**3.**	Welchen **höchsten** Schulabschluss hat **Ihr Partner/Ihre Partnerin**?<br>☐ Hauptschule/Volksschule<br>☐ Realschule/Mittlere Reife<br>☐ Polytechnische Oberschule<br>☐ Fachhochschulreife<br>☐ Abitur/allgemeine Hochschulreife<br>☐ anderen Schulabschluss, welchen: ....<br>☐ keinen Schulabschluss

**4.**	Wenn Sie wirklich Hilfe und Unterstützung brauchen, auf wen können Sie sich verlassen? (Bei dieser Frage sind mehrere Angaben möglich)<br>☐ Partner/Partnerin<br>☐ Kinder<br>☐ Eltern/Schwiegereltern<br>☐ sonstige Verwandte<br>☐ Nachbarn<br>☐ Freund/Freundin<br>☐ andere, z.B. Kollegen<br>☐ niemanden

**5.**	Wie viele Freunde oder Verwandte haben Sie, mit denen Sie sich *eng* verbunden fühlen und mit denen Sie auch persönliche Probleme besprechen können?<br>|__|__| (bitte Anzahl eintragen)

6. Wie würden Sie Ihre **Zufriedenheit im privaten Bereich** (Familie, Freizeit) in verschiedenen Lebensabschnitten beurteilen?
Bitte kreuzen Sie für jede Altersstufe bis einschließlich Ihrer jetzigen Altersstufe die am ehesten zutreffende Kategorie an.

	**sehr zufrieden**	**eher zufrieden**	**eher unzufrieden**	**sehr unzufrieden**
20-29 Jahre	☐	☐	☐	☐
30-39 Jahre	☐	☐	☐	☐
40-49 Jahre	☐	☐	☐	☐
50-59 Jahre	☐	☐	☐	☐
60-70 Jahre	☐	☐	☐	☐
älter als 70 Jahre	☐	☐	☐	☐

**7.**	Wie viele Personen leben derzeit ständig in Ihrem Haushalt, Sie selbst eingeschlossen?<br><br>|__|__| (bitte Anzahl eintragen)

**8.**	Die abschließende Frage nach Ihrem Einkommen wird selbstverständlich, ebenso wie alle anderen Angaben in diesem Fragebogen, streng vertraulich behandelt und nur vollständig anonym ausgewertet.<br><br>Wie hoch ist Ihr monatliches **Netto-Haushaltseinkommen,** d.h. das Nettoeinkommen, das Ihnen *allen zusammen* nach Abzug der Steuern und Sozialabgaben bleibt?<br><br>☐ weniger als 1.000 DM<br>☐ über 1.000, bis zu 2.000 DM<br>☐ über 2.000, bis zu 3.000 DM<br>☐ über 3.000, bis zu 4.000 DM<br>☐ über 4.000, bis zu 5.000 DM<br>☐ über 5.000, bis zu 6.000 DM<br>☐ über 6.000, bis zu 7.000 DM<br>☐ über 7.000 DM

Für Ihre Mitarbeit möchten wir uns sehr herzlich bedanken!

Bitte geben Sie nun den ausgefüllten Fragebogen
bei einem unserer Studienmitarbeiter ab.

Möchten Sie uns noch etwas mitteilen, was Ihnen wichtig erscheint?
Auf folgenden Zeilen haben Sie Gelegenheit, uns Ihre Meinung mitzuteilen oder noch auf Punkte hinzuweisen, die im Fragebogen nicht berücksichtigt wurden:

....

....

....

....

....

....

....

....

LANGZEITERFOLGE DER ***KAR***DI***OL***OGISCHEN ***A***NSCHLUSSHEILBEHANDLUNG

Patientenfragebogen

- am Ende der Reha -

ID-Nummer:

Ausgabedatum: |__|__|.|__|__|.|__|__|__|__| (Tag.Monat.Jahr)

Rückgabe bis zum: |__|__|.|__|__|.|__|__|__|__|

Rückgabe am: |__|__|.|__|__|.|__|__|__|__|

Sehr geehrte Teilnehmerin, sehr geehrter Teilnehmer der ***KAROLA***-Studie!

Ihr Aufenthalt hier in der Klinik geht nun bald zu Ende. Wir hoffen, Sie haben sich in der Klinik wohlgefühlt und Ihren Gesundheitszustand Ihren Möglichkeiten und Erwartungen entsprechend verbessern können.

Zur Verbesserung von Rehabilitationsmaßnahmen ist es für uns auch sehr wichtig, etwas darüber zu erfahren, wie es Ihnen jetzt, am Ende Ihres stationären Aufenthalts, geht. Wir möchten Ihnen daher im folgenden einige Fragen zum Rehabilitationsverlauf und -ergebnis stellen, so wie sich der Aufenthalt aus Ihrer Sicht darstellt.

Der Fragebogen gliedert sich in die Abschnitte: Gesamteindruck, Ernährung, Gesundheitszustand und seelisches Befinden.

Bitte gehen Sie Frage für Frage sorgfältig durch und beantworten Sie alle Fragen vollständig. Sind Sie sich bei der einen oder anderen Frage nicht ganz sicher, so geben Sie bitte den aus Ihrer Sicht bestmöglichen Schätzwert an. Selbstverständlich werden alle Ihre Angaben streng vertraulich behandelt und ausschließlich in pseudonymisierter Form ausgewertet.

<u>So wird es gemacht:</u>

Beim Ausfüllen des Bogens machen Sie bitte in die vorgesehenen Kästchen jeweils ein Kreuz

z.B. ich fühle mich angespannt ☐ meistens ☒ gelegentlich ☐ nie

1. Ihr Gesamteindruck

1. Wie beurteilen Sie insgesamt den Erfolg Ihres stationären Aufenthalts in dieser Klinik?

☐ Ich bin insgesamt sehr zufrieden mit dem Rehabilitationsverlauf.
Meine Erwartungen wurden voll erfüllt

☐ Ich bin insgesamt zufrieden mit dem Rehabilitationsverlauf

☐ Ich bin insgesamt nicht besonders zufrieden mit dem Rehabilitationsverlauf

☐ Ich bin insgesamt unzufrieden mit dem Rehabilitationsverlauf

2. Wie zufrieden waren Sie mit den folgenden Angeboten/Behandlungen in der Klinik?

	**sehr zufrieden**	**zufrieden**	**nicht besonders zufrieden**	**unzufrieden**
ärztliche Betreuung	☐	☐	☐	☐
Betreuung durch Schwestern und Pfleger	☐	☐	☐	☐
Bewegungstraining	☐	☐	☐	☐
Vorträge/Seminare	☐	☐	☐	☐
Mahlzeiten-Angebot	☐	☐	☐	☐
Freizeit-Angebot	☐	☐	☐	☐

3. Haben Sie durch die Reha Anregungen (z.B. zur Ernährung oder zur Lebensweise) erhalten, die Sie zu Hause ausprobieren möchten?

**sehr viele**	**viele**	**wenige**	**keine**
☐	☐	☐	☐

2. Ihre Ernährung

1. Welche **Kostform** haben Sie während der Reha bekommen? Geben Sie bitte bei Diabetes Kost Ihre BE Zahl. (Es sind mehrere Antworten möglich)

☐ Vollkost ☐ Schonkost ☐ Reduktionskost

☐ cholesterinarme Kost ☐ Diabetes Kost (|__|__| BE) ☐ Vegetarische Kost

☐ Sonstiges

Haben Sie während der Reha **Fastentage** (z.B. nur Obst, nur Gemüse) eingelegt?

**ja, 1-2 Tage**	**ja, 3-4 Tage**	**ja, 5 oder mehr Tage**	**nein, nie**
☐	☐	☐	☐

2. Wie hat Ihnen das **Mittagessen** in der Regel geschmeckt?

**sehr gut**	**gut**	**nicht besonders gut**	**es hat mir nicht geschmeckt**
☐	☐	☐	☐

3. Für wie wichtig halten Sie die Ernährung für den weiteren Verlauf Ihrer Erkrankung?

**sehr wichtig**	**wichtig**	**nicht so wichtig**	**überhaupt nicht wichtig**
☐	☐	☐	☐

4. Wie viele **Zwischenmahlzeiten** (d.h. alle Mahlzeiten, Snacks, etc. zusätzlich zu den Hauptmahlzeiten) haben Sie während der Reha **täglich** im Durchschnitt zu sich genommen?

**mehr als 6**	**6**	**5**	**4**	**3**	**2**	**1**	**keine**
☐	☐	☐	☐	☐	☐	☐	☐

5.	Wie beurteilen Sie die Größe der einzelnen Portionen beim **Mittagessen** im Vergleich zu den gewohnten Portionen Ihrer Hauptmahlzeit (z.B. Mittag- oder Abendessen) zu Hause?			
		**eher kleiner als zu Hause**	**etwa gleich groß wie zu Hause**	**eher größer als zu Hause**
	Fleisch	☐	☐	☐
	Gemüse	☐	☐	☐
	Salat	☐	☐	☐
	Kartoffeln, Knödel, Nudeln, Spätzle, Reis	☐	☐	☐
	Soße	☐	☐	☐

6. Wie oft haben Sie die folgenden Nahrungsmittel während der Reha zu sich genommen? Denken Sie bitte bei der Beantwortung dieser Frage an alle Mahlzeiten, also an **Frühstück, Mittagessen, Abendessen** und an alle **Zwischenmahlzeiten**.

Bitte geben Sie für jede Lebensmittelgruppe an, wie viele Portionen Sie im Durchschnitt davon verzehrt haben und in welchem Zeitraum Sie die angegebene Anzahl Portionen verzehrt haben. Im folgenden zeigen wir Ihnen in 3 möglichen Beispielen, wie Sie die Eintragungen vornehmen sollten:

Beispiel 1: Während der Reha haben Sie im Durchschnitt morgens 2 Scheiben Graubrot und abends 1 Scheibe Graubrot gegessen, also insgesamt 3 Scheiben am Tag. Dann tragen Sie bitte unter **Anzahl der Portionen** *3* ein und kreuzen **pro Tag** an (siehe unten).

Beispiel 2: Sie haben ungefähr 2x in der Woche, jeweils 1 Becher Joghurt gegessen, also insgesamt 2 Becher Joghurt in der Woche. Dann tragen Sie bitte unter **Anzahl der Portionen** *2* ein und kreuzen **pro Woche** an (siehe unten).

Beispiel 3: Sie haben einmal während der gesamten Reha 1 Glas Wein getrunken. Dann tragen Sie bitte unter **Anzahl der Portionen** *1* ein und kreuzen **während der gesamten Reha** an (siehe unten).

Beispiele (1-3)	**Anzahl der Portionen, Gläser, etc.**	**In welchem Zeitraum?** **pro Tag**	**pro Woche**	**während der gesamten Reha**	**nie**
1. Brot, Brötchen (1 Scheibe bzw. 1 Brötchen)	|__|3_|	☒	☐	☐	☐
2. Quark, Joghurt, Dickmilch, Buttermilch (1 Becher,ca.150 g)	|__|2_|	☐	☒	☐	☐
3. Wein, Sekt, Obstwein (Most) (1 Glas, ca. 0,25 l)	|__|1_|	☐	☐	☒	☐

Fortsetzung Frage ***6***	**Anzahl der Portionen, Gläser, etc.**	**In welchem Zeitraum?** **pro Tag**	**pro Woche**	**während der gesamten Reha**	**nie**
Fleisch und Wurstwaren					
Fleisch inkl. Geflügel, ohne Wurstwaren (1 Portion, ca.150 g)	|__|__|	☐	☐	☐	☐
magere Wurst (z.B. Geflügel-wurst, gekochter Schinken, etc.) (1 Scheibe bzw. 1 Portion)	|__|__|	☐	☐	☐	☐
sonstige Wurst (1 Scheibe bzw. 1 Portion)	|__|__|	☐	☐	☐	☐
Fisch (1 Portion, ca. 200 g)	|__|__|	☐	☐	☐	☐
Beilagen					
Salzkartoffeln, Knödel (1 Portion)	|__|__|	☐	☐	☐	☐
Pommes frites, Kroketten, Bratkartoffeln (1 Portion)	|__|__|	☐	☐	☐	☐
Nudeln, Spätzle (1 Portion)	|__|__|	☐	☐	☐	☐
Reis, Grünkern, Hirse, etc. (1 Portion)	|__|__|	☐	☐	☐	☐
Eier, Milch, Milchprodukte					
Eier (1 Ei)	|__|__|	☐	☐	☐	☐
Milch, Kakao (1 Glas, ca. 0,2 l)	|__|__|	☐	☐	☐	☐
Halbfettmargarine (1 Portion, ca. 10 g)	|__|__|	☐	☐	☐	☐
Butter, sonstige Margarine (1 Portion, ca. 10 g)	|__|__|	☐	☐	☐	☐
Quark, Joghurt, Dickmilch, Buttermilch (1 Becher, ca. 150 g)	|__|__|	☐	☐	☐	☐
geschlagene Sahne (1 Portion)	|__|__|	☐	☐	☐	☐
Käse mit reduziertem Fett-gehalt (1 Scheibe bzw. 1 Portion)	|__|__|	☐	☐	☐	☐
sonstiger Käse (1 Scheibe bzw. 1 Portion)	|__|__|	☐	☐	☐	☐

*Fortsetzung Frage **6***

	Anzahl der Portionen, Gläser, etc.	In welchem Zeitraum? pro Tag	pro Woche	während der gesamten Reha	nie
Obst und Gemüse					
Frisches Obst (1 Stück bzw. 1 Portion)	|__|__|	☐	☐	☐	☐
Salat oder Gemüse, roh (1 Portion)	|__|__|	☐	☐	☐	☐
Gemüse, gekocht (1 Portion)	|__|__|	☐	☐	☐	☐
Getreideerzeugnisse					
Vollkornbrot, -brötchen (1 Scheibe bzw. 1 Brötchen)	|__|__|	☐	☐	☐	☐
Brot, Brötchen (1 Scheibe bzw. 1 Brötchen)	|__|__|	☐	☐	☐	☐
Haferflocken, Müsli, Cornflakes (1 Portion)	|__|__|	☐	☐	☐	☐
Süßwaren					
Pudding, Kompott, Eis (1 Portion)	|__|__|	☐	☐	☐	☐
Marmelade, Honig (1 Teelöffel)	|__|__|	☐	☐	☐	☐
Zucker (1 Teelöffel)	|__|__|	☐	☐	☐	☐
Schokolade, Pralinen, Schokoriegel (1 Riegel bzw. vergleichbare Menge)	|__|__|	☐	☐	☐	☐
Kuchen, Gebäck, Kekse (1 Kuchenstück bzw. vergleichbare Menge)	|__|__|	☐	☐	☐	☐
Salzige Esswaren					
salzige Knabbereien (z.B. Chips) (1 Portion, ca. 100g)	|__|__|	☐	☐	☐	☐
Fertiggerichte (z.B. Suppen, Pizza, Nudelgerichte) (1 Port.)	|__|__|	☐	☐	☐	☐

Fortsetzung Frage ***6***

	Anzahl der Portionen, Gläser, etc.	In welchem Zeitraum? pro Tag	pro Woche	während der gesamten Reha	nie
Alkoholfreie Getränke					
Mineralwasser (1 Glas, ca. 0,2 l)	|__|__|	☐	☐	☐	☐
Kalorienreduzierte Getränke (z.B. Cola light 1 Glas, ca. 0,2 l)	|__|__|	☐	☐	☐	☐
sonstige Erfrischungsgetränke (z.B. Obstsaft, Cola, ca. 0,2 l)	|__|__|	☐	☐	☐	☐
Kaffee, Tee					
Kaffee (1 Tasse)	|__|__|	☐	☐	☐	☐
Tee (1 Tasse)	|__|__|	☐	☐	☐	☐
Alkohol					
Bier (1 Flasche, 0,5 l)	|__|__|	☐	☐	☐	☐
Wein, Sekt, Obstwein (Most) (1 Glas, ca. 0,25 l)	|__|__|	☐	☐	☐	☐
Spirituosen (Schnaps, Likör, Weinbrand, u.ä./1 Glas, ca. 2 cl)	|__|__|	☐	☐	☐	☐

7. An welchen Veranstaltungen oder Beratungen zum Thema Ernährung haben Sie während der Reha regelmäßig teilgenommen?

☐ Vorträge zu gesunder Ernährung

☐ Einzelberatung

☐ Diabetikerschulung

☐ Lehrküche, Kochkurs

☐ ich habe an keinen Veranstaltungen zum Thema Ernährung teilgenommen

3. Ihr Gesundheitszustand

1. Beurteilen Sie bitte Ihren aktuellen Gesundheitszustand **jetzt, am Ende der Reha** anhand der folgenden Fragen:

	**gar nicht**	**sehr leicht**	**leicht**	**mäßig**	**stark**	**sehr stark**
Haben Sie Schmerzen im Kreuz, im Nacken oder im Rücken?	☐	☐	☐	☐	☐	☐
Haben Sie Gelenkbeschwerden in den Schultern, Armen oder Händen?	☐	☐	☐	☐	☐	☐
Haben Sie Gelenkbeschwerden in den Hüften, Beinen oder Füßen?	☐	☐	☐	☐	☐	☐
Haben Sie Schmerzen in der Brust oder Herzgegend?	☐	☐	☐	☐	☐	☐
Werden Sie kurzatmig, wenn Sie bergan oder schnell gehen?	☐	☐	☐	☐	☐	☐
Haben Sie ein Druck- oder Völlegefühl im Leib?	☐	☐	☐	☐	☐	☐
Gibt es bei Ihnen Stuhlgangprobleme?	☐	☐	☐	☐	☐	☐
Leiden Sie tags- oder nachtsüber unter Husten?	☐	☐	☐	☐	☐	☐
Leiden Sie unter Schlafstörungen?	☐	☐	☐	☐	☐	☐
Fühlen Sie sich erschöpft?	☐	☐	☐	☐	☐	☐
Fühlen Sie sich innerlich angespannt und nervös?	☐	☐	☐	☐	☐	☐
Fühlen Sie sich entmutigt und traurig?	☐	☐	☐	☐	☐	☐

2.	Im folgenden sind einige Tätigkeiten beschrieben, die Sie vielleicht an einem normalen Tag ausüben. Fühlen Sie sich durch Ihren derzeitigen Gesundheitszustand bei diesen Tätigkeiten eingeschränkt? Wenn ja, wie stark?

	**ja, stark einge-schränkt**	**ja, etwas einge-schränkt**	**nein, überhaupt nicht einge-schränkt**
Anstrengende Tätigkeiten, z.B. schnell laufen, schwere Gegenstände heben, anstrengenden Sport treiben	☐	☐	☐
Mittelschwere Tätigkeiten, z.B. einen Tisch verschieben	☐	☐	☐
Einkaufstaschen heben oder tragen	☐	☐	☐
Mehrere Treppenabsätze steigen	☐	☐	☐
Einen Treppenabsatz steigen	☐	☐	☐
Sich beugen, knien, bücken	☐	☐	☐
Mehr als 1 Kilometer zu Fuß gehen	☐	☐	☐
Mehrere Straßenkreuzungen weit zu Fuß gehen	☐	☐	☐
Eine Straßenkreuzung weit zu Fuß gehen	☐	☐	☐
Sich baden oder anziehen	☐	☐	☐

3.	Wie beurteilen Sie insgesamt Ihr Leistungsvermögen im Augenblick?

**sehr schlecht**	**schlecht**	**weniger gut**	**zufrieden-stellend**	**gut**	**sehr gut**
☐	☐	☐	☐	☐	☐

4. Ihre derzeitigen alltäglichen Sorgen und Probleme:

	**gar nicht**	**sehr leicht**	**leicht**	**mäßig**	**stark**	**sehr stark**
Machen Sie sich Sorgen um Ihre Gesundheit?	☐	☐	☐	☐	☐	☐
Falls berufstätig: Wie sehr sorgen Sie sich, aus gesundheitlichen Gründen Ihren Beruf aufgeben zu müssen?	☐	☐	☐	☐	☐	☐
Falls berufstätig: Tragen Sie sich zur Zeit mit dem Gedanken, sich vorzeitig berenten zu lassen?	☐ ja	☐ nein				

5. Aus Ihrer jetzigen Sicht:
Wie würden Sie rückblickend Ihren Gesundheitszustand **am Anfang der Reha** beurteilen?

**sehr schlecht**	**schlecht**	**weniger gut**	**zufrieden-stellend**	**gut**	**sehr gut**
☐	☐	☐	☐	☐	☐

Wie würden Sie Ihren Gesundheitszustand jetzt **am Ende der Reha** beurteilen?

**sehr schlecht**	**schlecht**	**weniger gut**	**zufrieden-stellend**	**gut**	**sehr gut**
☐	☐	☐	☐	☐	☐

4. Ihr seelisches Befinden

Bitte beantworten Sie die folgenden Fragen (Nr. 1-14) so, wie es für Sie persönlich **in der letzten Woche** am ehesten zutraf. Machen Sie nur ein Kreuz pro Frage und lassen Sie bitte keine Frage aus. Überlegen Sie bitte nicht lange, sondern wählen Sie die Antwort aus, die Ihnen auf Anhieb am zutreffendsten erscheint.

1.	**Ich fühle mich angespannt oder überreizt**<br>☐ meistens<br>☐oft<br>☐ von Zeit zu Zeit/gelegentlich<br>☐ überhaupt nicht
2.	**Ich kann mich heute noch so freuen wie früher**<br>☐ ganz genau so<br>☐ nicht ganz so sehr<br>☐ nur noch ein wenig<br>☐ kaum oder gar nicht
3.	**Mich überkommt eine ängstliche Vorahnung, dass etwas Schreckliches passieren könnte**<br>☐ ja, sehr stark<br>☐ ja, aber nicht allzu stark<br>☐ etwas, aber es macht mir keine Sorgen<br>☐ überhaupt nicht
4.	**Ich kann lachen und die lustige Seite der Dinge sehen**<br>☐ ja, so viel wie immer<br>☐ nicht mehr ganz so viel<br>☐ inzwischen viel weniger<br>☐ überhaupt nicht

5.	**Mir gehen beunruhigende Gedanken durch den Kopf**<br>☐ einen Großteil der Zeit<br>☐ verhältnismäßig oft<br>☐ von Zeit zu Zeit, aber nicht allzu oft<br>☐ nur gelegentlich/nie
6.	**Ich fühle mich glücklich**<br>☐ überhaupt nicht<br>☐ selten<br>☐ manchmal<br>☐ meistens
7.	**Ich kann behaglich dasitzen und mich entspannen**<br>☐ ja, natürlich<br>☐ gewöhnlich schon<br>☐ nicht oft<br>☐ überhaupt nicht
8.	**Ich fühle mich in meinen Aktivitäten gebremst**<br>☐ fast immer<br>☐ sehr oft<br>☐ manchmal<br>☐überhaupt nicht
9.	**Ich habe manchmal ein ängstliches Gefühl in der Magengegend**<br>☐ überhaupt nicht<br>☐ gelegentlich<br>☐ ziemlich oft<br>☐ sehr oft

10.	**Ich habe das Interesse an meiner äußeren Erscheinung verloren**<br>☐ ja, stimmt genau<br>☐ ich kümmere mich nicht so sehr darum, wie ich sollte<br>☐ möglicherweise kümmere ich mich zu wenig darum<br>☐ ich kümmere mich so viel darum wie immer
11.	**Ich fühle mich rastlos, muß immer in Bewegung sein**<br>☐ ja, tatsächlich sehr<br>☐ ziemlich<br>☐ nicht sehr<br>☐ überhaupt nicht
12.	**Ich blicke mit Freude in die Zukunft**<br>☐ ja, sehr<br>☐ eher weniger als früher<br>☐ viel weniger als früher<br>☐ kaum bis gar nicht
13.	**Mich überkommt plötzlich ein panikartiger Zustand**<br>☐ ja, tatsächlich sehr oft<br>☐ ziemlich oft<br>☐ nicht sehr oft<br>☐ überhaupt nicht
14.	**Ich kann mich an einem guten Buch, einer Radio- oder Fernsehsendung freuen**<br>☐ oft<br>☐ manchmal<br>☐ eher selten<br>☐ sehr selten

Erinnern Sie sich sonst noch an etwas während Ihres Aufenthaltes hier in der Klinik, was Ihnen wichtig erscheint, im Fragebogen aber nicht berücksichtigt wurde?

Auf folgenden Zeilen haben Sie Gelegenheit, uns Ihre Meinung mitzuteilen oder noch auf Punkte hinzuweisen, die im Fragebogen nicht berücksichtigt wurden:

....

....

....

....

....

....

Bitte geben Sie nun den ausgefüllten Fragebogen
bei einem unserer Studienmitarbeiter ab.

Wir bedanken uns sehr herzlich für Ihre Mitarbeit und

wünschen Ihnen für Ihre Gesundheit alles Gute!
